# Biogenesis of RNase P RNA from an intron requires co-assembly with cognate protein subunits

**DOI:** 10.1093/nar/gkz572

**Published:** 2019-07-09

**Authors:** Geeta Palsule, Venkat Gopalan, Amanda Simcox

**Affiliations:** 1 Department of Molecular Genetics, The Ohio State University, Columbus, OH 43210, USA; 2 Center for RNA Biology, The Ohio State University, Columbus, OH 43210, USA; 3 Department of Chemistry and Biochemistry, The Ohio State University, Columbus, OH 43210, USA

## Abstract

RNase P RNA (RPR), the catalytic subunit of the essential RNase P ribonucleoprotein, removes the 5′ leader from precursor tRNAs. The ancestral eukaryotic RPR is a Pol III transcript generated with mature termini. In the branch of the arthropod lineage that led to the insects and crustaceans, however, a new allele arose in which RPR is embedded in an intron of a Pol II transcript and requires processing from intron sequences for maturation. We demonstrate here that the *Drosophila* intronic-RPR precursor is trimmed to the mature form by the ubiquitous nuclease Rat1/Xrn2 (5′) and the RNA exosome (3′). Processing is regulated by a subset of RNase P proteins (Rpps) that protects the nascent RPR from degradation, the typical fate of excised introns. Our results indicate that the biogenesis of RPR *in vivo* entails interaction of Rpps with the nascent RNA to form the RNase P holoenzyme and suggests that a new pathway arose in arthropods by coopting ancient mechanisms common to processing of other noncoding RNAs.

## INTRODUCTION

RNase P is an essential endoribonuclease that is required to cleave the 5′ leader of precursor tRNAs (1–3). The ribonucleoprotein (RNP) form of RNase P is widespread in all three domains of life (1–3). The RNP consists of a catalytic RNA (RNase P RNA, RPR) and a variable number of protein subunits (RNase P proteins, Rpps). The number of Rpps associated with the RPR increases from one in bacteria, to four or five in archaea, and eight to ten in eukaryotes (3–5). In archaea and eukaryotes, the additional Rpps appear to compensate for structural features present only in bacterial RPRs (2,3). Biochemical reconstitution (6–8) and high-resolution structural (9–11) studies of RNase P from all three domains of life shed light on protein-aided RNA catalysis in this ancient RNP enzyme. However, little is known about the sequence of assembly events *in vivo* for the multi-subunit eukaryotic RNase P holoenzyme. Here, we report the discovery of a critical role for select Rpps in *Drosophila* RPR biogenesis.

In *Drosophila*, a representative of a large group of animals, including the insects and crustaceans, *RPR* is embedded in an intron of a protein-coding recipient gene and transcribed by Pol II as part of the recipient gene transcript (12). Prior to our analysis of *Drosophila* RPR, other eukaryotic RPRs, ranging from yeast to human, were demonstrated to be independently transcribed Pol III-regulated genes (13–17). With the exception of budding yeast (15,18), the canonical Pol III-regulated RPRs are generated with their mature termini and do not require additional processing for maturation. In contrast, the embedded form of RPR, as exemplified by *Drosophila*, must be processed by nucleases from an intron thus necessitating a distinct mode of biogenesis.

Here, we provide genetic evidence that the biogenesis of *Drosophila* intronic RPR depends on splicing and subsequent exonucleolytic trimming of the 5′ and 3′ terminal nucleotides of the precursor-RPR (pre-RPR) by Rat1/Xrn2 and the RNA exosome, respectively. We further demonstrate that RPR biogenesis requires specific Rpps to protect the RPR from degradation. Parallels with the biogenesis of other non-coding RNAs, such as intronic small nucleolar RNAs (snoRNAs) (18–20), provides insights into the origin of this new mode for RPR biogenesis in an ancestor of the insects and crustaceans. It also raises questions about why the new *RPR* allele, which requires a more complex biogenesis pathway, was fixed in the descendants that currently comprise the most successful group of animals.

## MATERIALS AND METHODS

### Experimental model and subject details

Cell culture experiments were performed using *Drosophila melanogaster* S2 cells, which are embryonic and characterized as male. S2 cells were maintained in Schneider's medium (Sigma or Gibco) + 10% (v/v) heat inactivated fetal bovine serum + 0.1% (v/v) penicillin–streptomycin solution (Thermo Fisher Scientific). All cell culture experiments were conducted at 25°C. Further information about S2 cells can be found at https://dgrc.bio.indiana.edu/product/View?product=6 and elsewhere (21).

### Cell culture and RNAi-mediated knockdown

RNAi-mediated knockdown of gene expression was performed by bathing S2 cells with the *in vitro* synthesized, target-specific dsRNAs in serum-free media following a published protocol (22). For RNAi-mediated knockdown of each target gene (with the exception of Dis3 and Rrp6), two rounds of bathing for 72 h each with 20–30 μg of dsRNA were performed. For experiments involving transgenes, a 1-day transfection was conducted between the two rounds of dsRNA treatments. For Dis3 and Rrp6, the cells were treated with dsRNA by bathing for 48 h followed by transfection because we determined that longer incubations were detrimental to cell viability. All transfections were carried out using a Qiagen Effectene transfection kit according to the manufacturer's protocol.

### Generation of dsRNA for knockdown experiments

Genomic DNA was used as the template to generate amplicons in the range of 250–600 bp. Gene-specific target dsDNA sequences were identified using the *Drosophila* RNAi screening center (DRSC) database. PCR fragments containing the targeted sequence and the T7 promoter sequence were amplified for each gene of interest using gene-specific primers also containing the T7 promoter sequence at the 5′ end (see Supplementary Table S2). These PCR amplicons were *in vitro* transcribed into dsRNA using a Hi-Scribe (New England Biolabs, NEB) *in vitro* transcription (IVT) reaction kit. The IVTs were performed at 37°C for ∼16 h. The IVT reactions were treated with DNase I for 30 min at 37°C, and the RNA transcripts purified using a Direct-zol RNA miniprep kit (Zymo Research). Cells in one well of a six-well plate were bathed with 20–30 μg of dsRNA.

### RNA extraction

Total RNA was extracted from cell pellets resuspended in 50 μl of 1× PBS. One ml of Trizol reagent (Thermo Fisher Scientific) was added to the 50-μl cell suspension, vortexed vigorously, and was incubated at 22°C for 10 min. Subsequently, 200 μl of chloroform (Sigma-Aldrich) per 1 ml of Trizol (v/v) was added to the Trizol-cell mixture, vortexed vigorously, and incubated at 22°C for 10 min. The sample was centrifuged at 14 000 g for 15 min at 4°C. The aqueous phase was collected and an equal volume of 100% (v/v) ethanol was added, and the RNA was precipitated using the Zymo Direct-zol™ RNA MiniPrep kit as per the manufacturer's protocol.

### Reverse transcription (RT)-PCR and RT-qPCR

To prepare cDNA, a reverse transcription (RT) reaction was carried out using a universal olido(dT) reverse primer (Thermo Fisher Scientific) and the Omniscript RT kit (Qiagen). The efficiency of knockdown was detected by RT-PCR and/or RT-qPCR (see Supplementary Table S3 for primer sequences). qPCR was performed using a SYBR green PCR mix (Thermo Fisher Scientific) on an Applied Biosystem qPCR instrument.

### Northern blots

**Figure 1.**
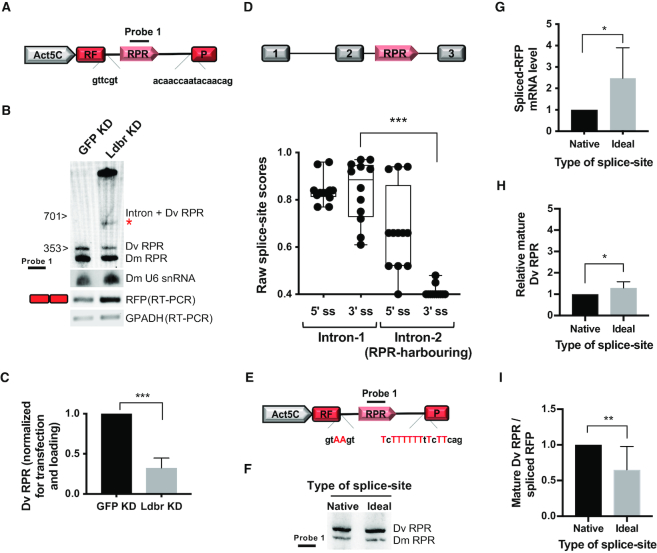
Perturbing splicing of the recipient intron affects RPR maturation. (**A**) Split-RFP reporter. Act5C promoter (Pol II), grey; *RFP* exons, red; *Drosophila virilis* intron-encoding *RPR*, pink; splice sites (native), shown in lower case; Probe 1, RPR antisense probe. Intron, 701 nt; RPR, 353 nt. (See also Supplementary Table S1 for additional details on probes used). (**B**) Northern blot of RNA extracted from S2 cells treated with dsRNA to knockdown either lariat debranching enzyme (Ldbr KD) or GFP control (GFP KD) and transfected with the split-RFP reporter (Figure 1A). Asterisk, faster-migrating intermediate. *Drosophila melanogaster* (Dm) U6 snRNA, loading control for northern; RFP RT-PCR, RFP expression was used to assess transfection and splicing efficiency; GAPDH, loading control for RT-PCR. (**C**) Quantitation of northern blot in (B) shows that the level of Dv RPR is significantly decreased following Ldbr KD when compared to the control (GFP KD) (*n* = 3). (**D**) Schematic depicting the conserved location of *RPR* in the second intron of the *ATPsynC* gene in 12 sequenced *Drosophila* species. Box and whisker plot, showing donor (5′ splice-site, 5′ ss) and acceptor (3′ splice-site, 3′ ss) splice-site scores for the first and second intron in *ATPsynC*. Each dot represents the splice-site score for one of the 12 species (see also, Supplementary Figure S1D for scores in individual species). (**E**) Split-RFP reporter with ‘ideal splice-sites’. The split-RFP reporter was mutated to incorporate canonical (strong) *D. melanogaster* 5′ donor and 3′ acceptor splice sites. Mutations are depicted in red uppercase (see also Supplementary Figure S1D). (**F**) Northern blot of RNA extracted from S2 cells expressing *D. virilis* RPR from the reporter with native or ‘ideal splice-sites’ (A and E) (see also Supplementary Table S1). (**G**) Quantitation of the spliced-RFP mRNA in cells transfected with the ‘native’ (A) or ‘ideal splice-site’ (E) reporters. Gene expression was determined by RT-qPCR; RFP data normalized to GAPDH mRNA (*n* = 8). (**H, I**) Quantitation of Dv RPR expression in cells transfected with the ‘native’ (A) or ‘ideal’ (E) splice-site reporters; Dv RPR normalized to either Dm RPR (H) or RFP (I) expression (*n* = 8). Note: Significance values are *P* values, calculated by an unpaired *t* test. (G, H and I) or Wilcoxon's test (D): *, <0.05; **, <0.01; ***, <0.001. Error bars indicate standard deviation of the mean. (Supplementary Figure S1 and Supplementary Tables S1–S3 provide sequences of probes and primers.)

**Figure 2.**
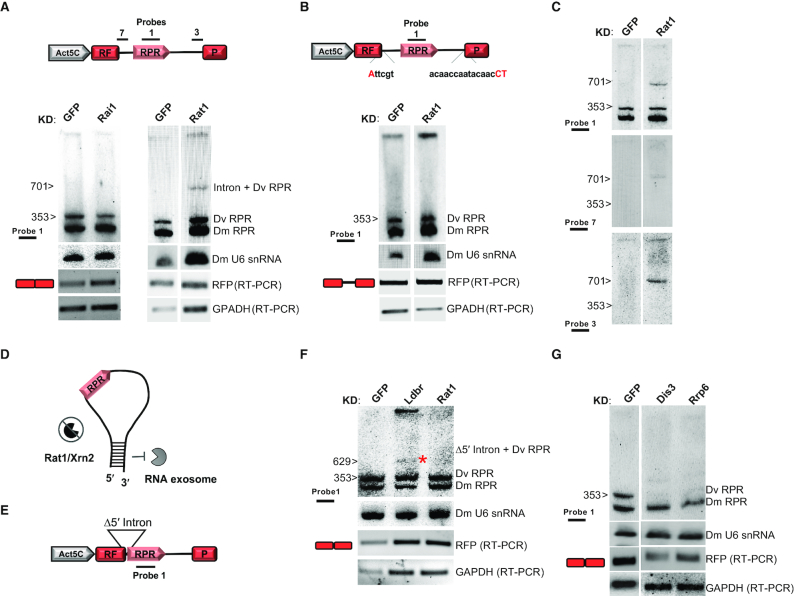
The spliced pre-RPR is trimmed by exonucleases Rat1/Xrn2 and the RNA exosome to produce mature RPR. (A and B) Northern blot of RNA extracted from S2 cells depleted for Rai1 (Rai1 KD) or Rat1/Xrn2 (Rat1 KD) or GFP (control, GFP KD) and transfected with a split RFP reporter with native (**A**) or mutant splice sites (**B**). Hybridization with Probe 1 shows an RPR intermediate corresponding in size to the Dv intron-containing RPR (Intron + DV RPR) only in cells with depleted levels of Rat1/Xrn2 and expressing the split-RFP reporter with native splice sites (A). Depletion of Rai1 has no observable effect on RPR maturation from the reporter. The intermediate is absent in the split-RFP reporter with non-functional splice sites (B). The *D. virilis* RPR intermediate recovered following Rat1/Xrn2 KD is the full-size intron as determined by comparison with an IVT-generated intron sequence (see also, Supplementary Figure S2A and Supplementary Table S1 for sequence of probes). (**C**) Northern blot shown in (A) hybridized with additional probes. Probes for RPR (Probe 1), the distal 5′ intron sequence (Probe 7), and the distal 3′ intron sequence (Probe 3) hybridize to an intermediate following RNAi against Rat1/Xrn2. (**D**) Model depicting a predicted secondary structure formed by RPR-flanking intron sequences when 5′ processing is ablated (Rat1/Xrn2 depletion), which potentially blocks 3′ processing (see also, Supplementary Table S4 for sequence of split-RFP reporter with *D. virilis* intron, with native splice-sites, containing RPR). (**E**) Split-RFP reporter (Figure 1A) with 5′-intron deletion (Δ = 72 nt) (see also Supplementary Table S4). (**F**) Northern blot of RNA extracted from S2 cells depleted for Ldbr (Ldbr KD), Rat1/Xrn2 (Rat1 KD), or GFP (control, GFP KD) and transfected with a split RFP reporter with the 5′-intron deletion (Δ5′ intron). No intermediate corresponding to the Dv intron-containing RPR (Intron + DV RPR) is observed following Rat1/Xrn2 KD. A smaller intermediate is observed in the Ldbr KD sample (asterisk), indicating that splice sites are functional. Probe 1 (A) (see also, Supplementary Figure S2G and S2H, Supplementary Table S1–3). (**G**) Northern blot of RNA extracted from S2 cells depleted for Dis3 (Dis3 KD), Rrp6 (Rrp6 KD), or GFP (control, GFP KD) and transfected with a split RFP reporter (A). Depletion of the exosome nucleases, Dis3 or Rrp6, leads to a decrease in the level of mature DV RPR (see also Supplementary Figure S2B; Supplementary Table S1–S3). *D. melanogaster* (Dm) U6 snRNA, loading control for northern blots; RFP RT-PCR, RFP expression was used to assess transfection and splicing efficiency; GAPDH, loading control for RT-PCR (see also Supplementary Figure S2; Supplementary Table S1–S3).

**Figure 3.**
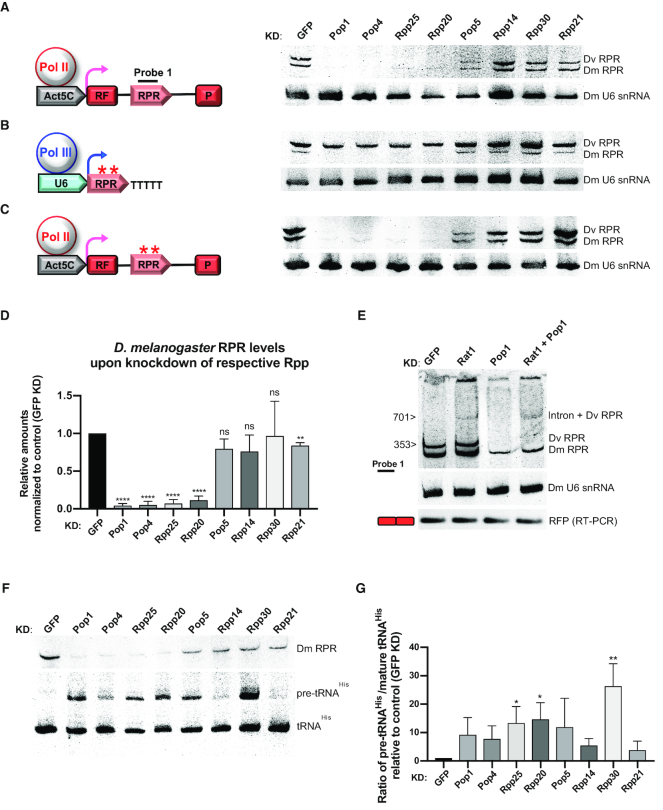
A subset of Rpps is required for *Drosophila* RPR maturation. (**A, B, C**: left) Constructs with *D. virilis RPR* expressed under regulation of either an Act5C-Pol II promoter (A and C) or a U6 snRNA-Pol III promoter (B). The red asterisks indicate point mutations (T53C, T55A, and T157C) that disrupt the two polythymidine (polyT) stretches (Pol III transcription termination signals) within the *D. virilis* RPR gene (B and C). (A, B, C: right) Northern blot analysis to determine RPR levels in S2 cells transfected with the indicated reporter and treated with dsRNA to knockdown GFP (control, GFP KD) or individual Rpps (Pop1, Pop4, Rpp25, Rpp20, Pop5, Rpp14, Rpp30 or Rpp21). (**D**) Quantitation of *D. melanogaster* RPR levels upon depletion of Rpps (Pop1, Pop4, Rpp25, Rpp20, Pop5, Rpp14, Rpp30 or Rpp21) normalized to GFP KD; *D. melanogaster* U6 snRNA, loading control (*n* = 3). (**E**) Northern analysis of RNA extracted from S2 cells, transfected with a split-RFP reporter (A) and treated with dsRNA to knockdown Rat1/Xrn2 (Rat1 KD) or Pop1 (Pop1 KD) or the combination (Pop 1 + Rat1 KD). The RPR intermediate observed following Rat1 KD or Pop1 + Rat1 KD combined, is absent following Pop1 KD. (**F**) Northern blot of RNA extracted from S2 cells after knockdown of individual Rpps. Total RNA was probed for *D. melanogaster* RPR (Dm RPR) (upper blot), pre-tRNA^His^ and mature tRNA^His^ (lower blot). (**G**) Ratio of pre-tRNA^His^/mature tRNA^His^ following KD of each Rpp relative to GFP KD (*n* = 3) (see also, Supplementary Figure S4 for quantitation of knockdowns). Significance values are *P* values, calculated by an unpaired *t* test (D and G): ns, not significant; *, <0.05; **, <0.01; ***, <0.001; ****, <0.0001. Error bars indicate standard deviation of the mean.

For gel electrophoresis, 10-20 μg of total RNA was separated on either an 8% (w/v) polyacrylamide + 7 M urea gel (Figures 1, 2 and 3A–C, E) or a 10% (w/v) polyacrylamide + 8 M urea gel (Figure 3F) and transferred to a nylon membrane (Hybond N^+^, GE Healthcare). Membranes were hybridized with non-radioactive, digoxygenin (DIG)-labelled DNA probes, which were generated using a terminal DIG-labelling kit (Sigma Aldrich Cat. #03353583910) (23). Blots were hybridized at 50°C for ∼16 h using an optimized protocol (23) (Roche, Sigma Aldrich Cat. #11585762001). The hybridized probe was detected using an anti-DIG antibody and the chemiluminescent substrate, CPD-Star. Due to the sequence similarity between the *D. virilis* and *D. melanogaster* RPR genes, both were detected using the RPR antisense Probe 1 (see also Figures 1B and F, 2A-C, G; Supplementary Table S1 for sequence details). A shorter *D. virilis-*specific RPR antisense probe (Dv RPR-specific, see Supplementary Table S1 for sequence), was also used in a mixture with Probe 1 for northern blot analysis in Figures 2F, 3A–C, E. The *D. melanogaster* RPR-specific antisense probe (see Supplementary Table S1 for sequence) was used for detecting endogenous RPR (used in northern blot detection, Figure 3F). Hybridization with a *D. melanogaster* U6 snRNA antisense probe was used to determine U6 snRNA levels as the loading control (Supplementary Table S1).

### Bioinformatics, quantitation and statistical analysis

Splice-site scores were predicted using the Berkley Drosophila Genome Project splicing algorithm specific to *Drosophila* (http://www.fruitfly.org/seq_tools/splice.html). The cut-off was set at 0.4 for a weak splice-site and 1.0 for a perfect splice-site. Wilcoxon's signed-rank test was performed to assess whether the means of the 3′ splice-site scores for the two introns in the *ATPsynC* gene in 12 *Drosophila* species are significantly different.

Northern blot quantitation in Figure 1C and H, Supplementary Figures S2B, S2C, S3A–D and S4F were quantitated using ImageJ software.

The expression of the nucleases or the Rpps following knockdown with dsRNAs was quantitated using qPCR, compared to the GFP control (GFP KD). The knockdown of expression is represented as mean ± SD, normalized to GAPDH mRNA. All the experiments reported in this study were repeated at least three times. The number of replicates (n) is indicated in the figure legends. Significance values are *P* values calculated using an unpaired *t* test or the Wilcoxon's test: *, <0.05; **, <0.01; ***, <0.001; ****, <0.0001. Error bars indicate standard deviation of the mean.

### Construction of plasmids and reporter genes

The relevant information is described in the supplementary material and Supplementary Tables S4 and S5.

## RESULTS

### Perturbing splicing of the recipient intron affects RPR maturation

To study if RPR maturation is influenced by splicing, we assayed the effects of either depletion of lariat debranching enzyme (Ldbr) or alteration of splice sites. In these experiments, we expressed a split-Red Fluorescent Protein (RFP) reporter gene with the *D. virilis* intron-encoding *RPR* inserted between two *RFP* exons (Dv RPR; Figure 1A). The larger size of *D. virilis* RPR (353 nucleotides, nt) allowed us to distinguish it from the endogenous RPR (302 nt) produced by the *D. melanogaster* S2 cells.

We tested *D. virilis* RPR generation in S2 cells that were depleted by RNAi of the Ldbr, an endoribonuclease that functions post-splicing to cleave the 2′-5′ phosphodiester bond of the intron lariat. Northern blot analysis of total RNA extracted from cells depleted of Ldbr led to accumulation of pre-RPR intermediates (Figure 1B), and a concomitant decrease in mature RPR synthesized from both the transgenic *D. virilis* and the endogenous *D. melanogaster* genes (Figure 1C and Supplementary Figure S1A). The effect on the expression of *D. virilis* RPR was more pronounced because, unlike the endogenous *D. melanogaster* RPR, the transgene was expressed only after Ldbr was depleted by RNAi.

In the absence of Ldbr, the expected intermediate is a branched lariat structure containing the RPR and flanking intron sequence. We observed two intermediates: a prominent slow-migrating species, which likely corresponds to the circular lariat, a structure known to retard electrophoretic mobility, or the pre-mRNA (20,24); a faster-migrating species, which is likely to correspond to a 3′-processed lariat or a nicked lariat. The presence of intron sequences in these lariat intermediates was confirmed by northern blot analysis with intron-specific antisense probes (Supplementary Figure S1B; Supplementary Table S1). The faster-migrating intermediate was smaller than the expected size of the full intron (701 nt) because it lacks the most distal part of the intron, 3′ to the branch site (Supplementary Figure S1B). Together, these results show that RPR biogenesis is adversely affected by Ldbr knockdown and is dependent on both splicing and debranching.

In the endogenous locus (12), *RPR* is in the second intron of the *ATPsynC* gene and we expect it to be produced in a splicing-dependent process as the recipient gene encodes an essential protein. Interestingly, however, the second intron in *ATPsynC*, which harbors *RPR*, has poor or absent 3′ splice-sites in all 12 annotated *Drosophila* species (Figure 1D and Supplementary Figure S1D). Unlike the second intron, the first intron in *ATPsynC* has normal splice-site scores (Figure 1D) making the gene an anomaly because introns in the same *Drosophila* gene usually exhibit low variation in splicing rates (25). To test for potential significance for these poor splice-sites in the RPR-containing intron, we constructed a reporter in which the native splice-sites (preserved in the original split-RFP reporter, Figure 1A), were mutated to create ‘ideal splice-sites’ matching the canonical splicing signals (Figure 1E). The ‘ideal splice-site’ reporter produced RFP and mature *D. virilis* RPR when transfected into S2 cells (Figure 1F) and led to an approximately 2.5-fold increase in spliced RFP mRNA in comparison to the ‘native splice-site’ reporter (Figure 1G). However, there was no concomitant proportionate increase in the level of mature RPR (Figure 1H), suggesting that RPR was processed less efficiently from the ‘ideal’ intron, even though the level of spliced mRNA had substantially increased. Collectively, these data suggest that inefficient splicing may favor RPR biogenesis. We hypothesize that the slower rate of splicing might allow more time for association with Rpps, including those that protect the nascent transcript from nuclease trimming. We describe below our efforts to identify these nucleases and the Rpps that afford protection from nucleolytic attack.

### Trimming of the 5′ leader of spliced pre-RPR is mediated by the exonuclease Rat1/Xrn2

Since production of mature *Drosophila* RPR requires processing to trim the 5′ and 3′ RPR-flanking intron sequences, we postulated a mode of biogenesis involving nucleases. To identify the 5′-processing exonuclease(s), we tested two ubiquitous, nuclear exonucleases Rai1 and Rat1/Xrn2. We used RNAi to deplete each exonuclease separately in S2 cells and assayed by northern blotting the effect of knockdown on *D. virilis* RPR maturation from the split-RFP reporter (Figure 2A). Depletion of Rat1/Xrn2, but not Rai1, led to accumulation of a pre-RPR intermediate with a concomitant decrease in mature RPR (Figure 2A and Supplementary Figures S2A, S3A–B). This intermediate was absent when Rat1/Xrn2 was depleted in cells expressing a split-RFP reporter with non-functional splice-sites, demonstrating that Rat1/Xrn2 functions post-splicing to process pre-RPR (Figure 2B). Northern blot analysis with probes spanning the intron showed that the pre-RPR intermediate contains sequences spanning the entire intron (Figure 2C and Supplementary Figure S2A; Supplementary Table S1). Our results are consistent with the fact that Rat1/Xrn2, but not Rai1, can process substrates with a 5′-monophosphate, which are generated by debranching of the lariat (26).

The presence of a full-length intron following Rat1/Xrn2 depletion suggested that 3′ processing was also unexpectedly affected in the absence of 5′ processing (Figures 2C and Supplementary Figure S2A). We hypothesized that this effect was likely indirect and that lack of Rat1/Xrn2-mediated 5′ trimming promotes formation of a 3′ processing-resistant secondary structure in the RPR-flanking intron sequence (Figure 2D; for intron sequence see Supplementary Table S4). To test this idea, we sought to eliminate this putative secondary structure by deleting 72 nt in the 5′ RPR-flanking intron of the split-RFP reporter (Figure 2E and Supplementary Table S4). The splice sites were intact as demonstrated by accumulation of a spliced-intron intermediate when the Ldbr enzyme was depleted (Figure 2F, Supplementary Figures S2G and H). However, depletion of Rat1/Xrn2 did not result in accumulation of an intermediate (Figure 2F). This observation is consistent with the idea that the 3′ processing machinery has access to the 3′ terminal nucleotides in the spliced intron provided these sequences are not sequestered in a secondary structure (pairing of 3′ and 5′ regions). This finding is reminiscent of snoRNA maturation where either secondary structure or association with snoRNP proteins dictates the extent of 5′ and 3′ trimming (19).

### Trimming of the 3′ end of spliced pre-RPR is mediated by the RNA exosome

To investigate the mechanism for pre-RPR 3′ processing, we tested a series of nucleases using RNAi in S2 cells expressing the reporter encoding *D. virilis* RPR (Figure 1A; Supplementary Tables S1 and S2). Knockdown of Rexo5 or Rex2 had no effect on RPR maturation (Supplementary Figures S2D–F), whereas depletion of the RNA exosome nucleases Dis3 or Rrp6 resulted in loss of mature *D. virilis* RPR (Figure 2G and Supplementary Figure S3C and D). Our results suggest that the RNA exosome, which functions in processing of snoRNAs (19,27) mRNAs (28), pre-rRNAs (29) and intronic-microRNAs (30) is also involved in pre-RPR processing. We observed complete loss of RPR rather than an intermediate, as seen following disruption of 5′ processing by Rat1/Xrn2 knockdown (Figure 2A). This observation suggests that failure to process the 3′ end may engender excessive 5′ trimming. However, this idea cannot be tested by combined Dis3/Rrp6 and Rat1/Xrn2 knockdown, as the latter knockdown results in a stable intermediate that is not susceptible to 3′ processing (Figure 2A).

### A subset of Rpps are required for protection during *Drosophila* RPR maturation

Since pre-mRNA introns are efficiently degraded post-splicing, any functional RNA encoded in an intron needs to be protected such that nuclease trimming only extends to the mature termini. For example, in the case of intronic snoRNAs, production of functional snoRNPs depends on recruitment of core snoRNP proteins during snoRNA maturation (31). However, since not all snoRNAs are obligatorily coupled to assembly with core snoRNA binding proteins (32), it was unclear if Rpps are critical for RPR maturation, a possibility that we investigated next.

We used RNAi in S2 cells to knockdown the eight predicted *Drosophila* Rpps (5) (Supplementary Figure S4; Supplementary Table S2) individually and examined accumulation of endogenous (*D. melanogaster*) and transgenic (*D. virilis*) RPRs by northern analysis (Figures 3A–C). Based on the effect of the individual Rpp depletion on RPR maturation, the Rpps split into two groups: depletion of Pop1 or Pop4 or Rpp25 or Rpp20 strongly decreased the levels of both the transgenic and endogenous RPRs, whereas, depletion of Pop5 or Rpp14 or Rpp30 or Rpp21 had little or no effect on the transgenic and the endogenous RPRs, when compared with the control (GFP KD) (Figures 3A–D). The decrease in level of both the transgenic and endogenous RPR suggests that depletion of Pop1 or Pop4 or Rpp25 or Rpp20 prevents generation of mature RPR and contributes to turnover of mature RPR. The basis for these thematic parallels remains to be uncovered.

Consistent with the idea that select Rpps protect RPR from nuclease attack, simultaneous depletion of Pop1 and Rat1/Xrn2 led to accumulation of an RPR intermediate, as seen upon Rat1/Xrn2 knockdown alone, whereas no intermediate was detected following Pop1 knockdown alone (Figure 3E and Supplementary Figure S5C).

To determine if this protective role of the Rpps in RPR biogenesis is required only when the intronic RPR is subject to nucleolytic trimming, we tested the effect of Rpp depletion on an RPR generated with its mature termini and as a Pol III transcript under the regulation of the U6 snRNA promoter. As the termination of Pol III transcription is signaled by polythymidine (polyT), we mutated two internal polyT stretches in the *D. virilis RPR* transgene to prevent potential premature termination of transcription (Supplementary Figures S5A and S5B; Supplementary Tables S4 and S5). In contrast to Pol II-regulated RPRs (endogenous *D. melanogaster* RPR and transgenic *D. virilis* RPR, Figure 3A), the Pol III-regulated U6-RPR was present when Pop1, Pop4, Rpp25 or Rpp20 were individually depleted (Figure 3B; see quantitation in Figure S4F). This finding suggests that an RPR transcribed with mature termini does not require association with Rpps for protection from nucleases, unlike the intron-derived, Pol II-transcribed RPRs that require nuclease mediated trimming for maturation. To rule out the possibility that the mutated polyT sequences in the Pol III-regulated transgene affected the outcome, we tested a Pol II variant with the mutated polyT sequences and found that it was affected similarly to a transgene encoding the native RPR sequence with respect to Rpp depletion (Figure 3A and C). This lack of Rpp dependence for RPR biogenesis was not related to the U6 promoter itself because this behavior was mirrored during RPR expression from a different Pol III promoter (Supplementary Figure S5D).

To test for RNase P activity following depletion of Rpps, we assayed tRNA maturation in S2 cells using northern blotting (33,34). As expected, individual depletion of the subunits (Pop1, Pop4, Rpp25 and Rpp20) required for RPR stability, also decreased RNase P activity as evidenced by accumulation of pre-tRNAs (Figure 3F and G). Individual depletion of Pop5, Rpp14, Rpp30 or Rpp21, which had little or no effect on RPR stability, also affected RNase P activity and showed accumulation of pre-tRNAs (Figure 3F and G). Rpp21 had the least effect on activity, consistent with the observation that *in vitro* reconstituted yeast RNase P did not require Rpp21 for activity (7).

Collectively, our *in vivo* data showed that all Rpps are required for the activity of RNase P, and further identified a subset that has a critical role in protecting the catalytic RNA during its maturation and prior to formation of the complete holoenzyme.

## DISCUSSION

### Splicing efficiency influences RPR biogenesis

In all insects and crustaceans examined, *RPR* is embedded in an intron of a protein- coding recipient gene (12). In *Drosophila* species, the RPR-containing intron has weak or missing acceptor splice-sites (Figure 1). Counterintuitively, there may be a link between poor splicing and more efficient RPR production, because a reporter gene with ‘ideal splice-sites’ did not give rise to substantially higher levels of the mature RPR (Figure 1). In the endogenous gene, however, splicing is required to produce the mRNA for the recipient gene, *ATPsynC*. But a slower splicing reaction for the second intron could be tolerated if sufficient levels of ATPsynC are generated, while also having the benefit of accommodating assembly of nascent RPR with Rpps to protect the RNA from nuclease attack after the lariat is debranched (Figure 3). It will be of interest to investigate the relative kinetics of splicing for the two introns in *ATPsynC* and to analyze the splice-site scores of other host introns with RPRs in various insects and crustaceans. A more widespread incidence of weak splice sites flanking RPR-harboring introns would support the idea that inefficient splicing is under positive selection.

**Figure 4.**
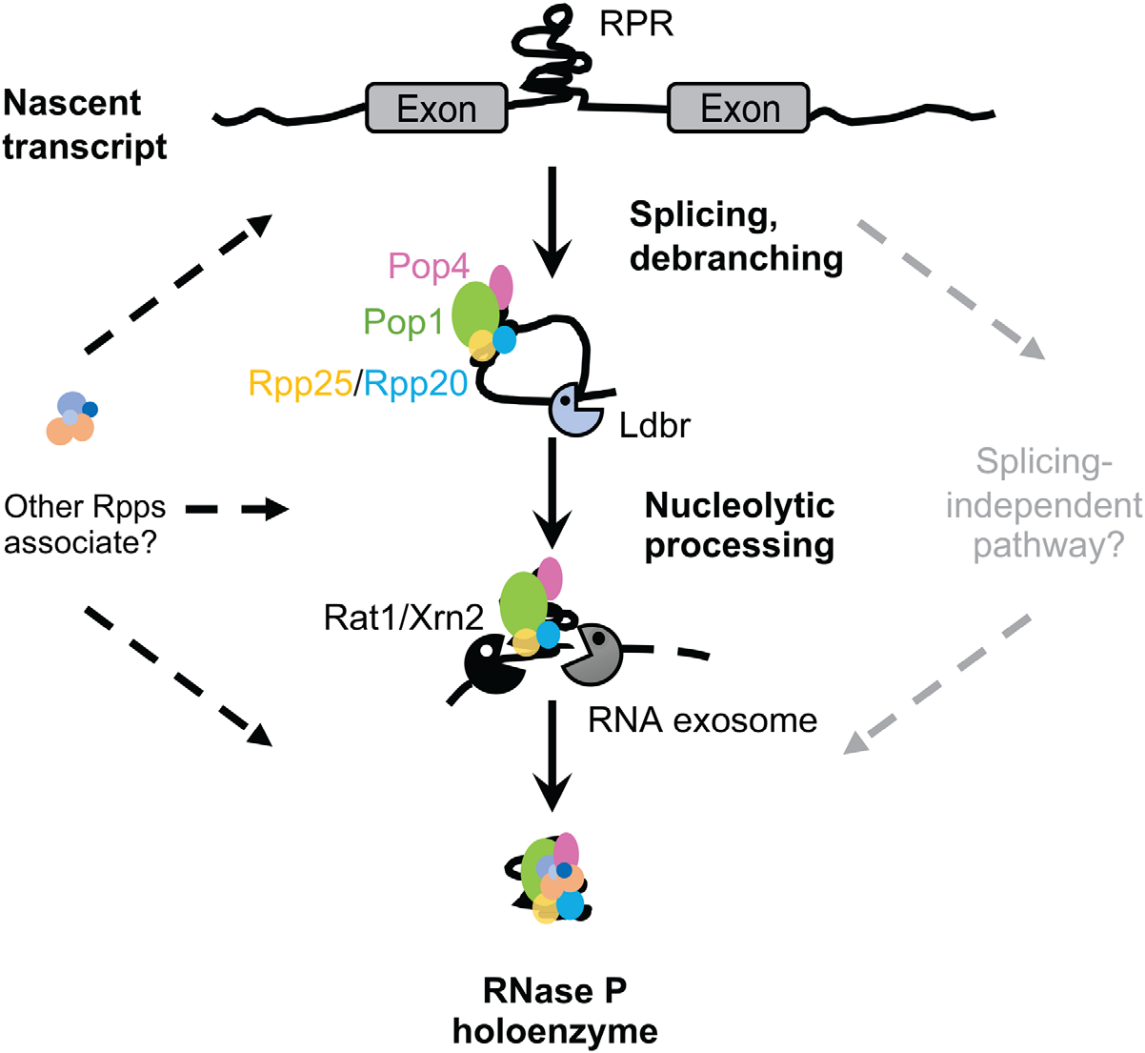
Model for biogenesis of intronic RPR in *Drosophila*. The nascent RPR transcript is spliced and debranched. The precursor RPR termini are processed by Rat1/Xrn2 and the RNA exosome. Protection from nucleolytic digestion beyond the mature termini is provided by Rpps (Pop 1, Rpp20, Rpp25 and Pop4 are shown, as RPR is degraded in their absence). Reporter-based assays (Figure 2B; and (12)) support an alternative splicing-independent pathway for RPR maturation (pathway depicted on the right); however, it remains to be established if this mechanism is used to aid RPR maturation from the endogenous locus.

Since RPR could be produced from an intron embedded in a reporter gene incapable of splicing (Figure 2B; and (12)), an alternative splicing-independent RPR biogenesis pathway might function in *Drosophila*. Presumably, processing in this case involved endonucleolytic cleavage of the unspliced intron followed by exonucleolytic trimming. Redundant pathways would enhance robustness in production of the essential RNase P enzyme (Figure 4). The use of splicing-dependent and -independent pathways with variable efficiencies is well documented for different yeast snoRNAs (32,35,36)

### Biogenesis of intronic RPR provides insights into holoenzyme formation *in vivo*

Like snoRNAs, biogenesis of RPR depends on processing by the conserved nucleases Rat1/Xrn2 and the RNA exosome (Figures 2–4) (19,37). This similarity extends to the finding that both RPR (Figures 2–4) and snoRNAs (19,38,39) require protection from the nucleases by association with their cognate protein subunits during synthesis. This parallel in RPR and snoRNA biogenesis suggests the generalization that functional ncRNAs can mature from an intron-embedded precursor through the action of non-specific nucleases provided protection against excessive nucleolytic trimming is afforded by cognate proteins.

RPR biogenesis requires association with a specific subset of Rpps: Pop1, Pop4, Rpp20 and Rpp25 (Figures 2–4). The importance of these Rpps can be rationalized based on the high-resolution structures of yeast and human RNase P and *in vitro* reconstitution of yeast RNase P (7,9,11). Pop1 (∼100 kDa protein) in yeast and human RNase P makes extensive contacts with the RPR and stabilizes the helical core of the RPR’s catalytic domain ((9,11) and Supplementary Figure S6). The intertwining of Pop1 with the RPR suggests that it may interact with the nascent RPR transcript and guide the RPR to its final fold (9,11). The 35-kDa heterodimer formed by Rpp20 and Rpp25 binds to the P3 helix of RPR ((9,11,40,41) and Supplementary Figure S6), and enhances the affinity of Pop1 for the RNA (42). Notably, *in vitro* reconstitution of yeast RNase P showed that Pop1, Rpp20 and Rpp25 can bind the RPR even without the other Rpps (7). The Rpp20/Rpp25 heterodimer binds first and chaperones Pop1 to initiate holoenzyme formation (7). Our *in vivo* results corroborate these *in vitro* assembly hierarchy data as we find Pop1, Rpp20 and Rpp25 are essential for RPR stability (Figures 3 and 4). We hypothesize that these Rpps assemble with nascent RPR, perhaps co-transcriptionally, and protect the RPR from nuclease attack that follows intron excision and lariat debranching (Figure 4).

RPR accumulation also decreased when Pop4 was depleted (Figure 3). Pop4, binds to the RPR’s substrate specificity domain (7) and serves as a bridge between this domain and the catalytic domain by binding other Rpps to generate a tightly interwoven RNP ((9,11) and Supplementary Figure S6). The absence of the molecular strut formed by Pop4 may lead to disassembly *in vivo* and expose the RPR to nucleolytic degradation. Pop4, which plays a role in interlocking other subunits, appears to be as critical as the primary RPR-binding RPPs.

We made the surprising discovery that the same Rpps were not required for stability when RPR was generated as a Pol III-transcript with mature termini, which would not require nucleolytic trimming (Figure 3). Yeast RPR, also a Pol III transcript, is susceptible to degradation in the absence of Pop1 (43). However, yeast RPR is made as a precursor and subject to nucleolytic trimming during maturation. Thus, we favor the idea that the dependence on Rpps for *Drosophila* RPR stability may not be dictated by the polymerase used for RPR transcription (Pol II or Pol III) but rather the need for protection during nucleolytic trimming of an RPR with immature termini.

### Conserved nucleases involved in *Drosophila* RPR biogenesis likely enabled the emergence of the new class of intronic RPRs in animals

The mode of *Drosophila* RPR biogenesis (Figure 4) fits well with a hypothetical origin of the intronic *RPR* which, at its inception approximately 500 million years ago in a common ancestor of insects and crustaceans, must have coopted ancient nucleases for processing. The Xrn family and the RNA exosome are strong candidates because they are conserved throughout eukaryotes and therefore predate birth of the intronic RPR (37,44). Moreover, the promiscuous activities of Rat1/Xrn2 and the RNA exosome are consistent with recruitment of new substrates such as the intronic RPR. We also assume that this nucleolytic processing was regulated by Rpps, which through their affinity for the ancestral Pol III RPR, would also have bound and protected nascent intronic RPR.

There may well have been a transition period when both alleles, ancestral Pol III-regulated and the new Pol II-regulated *RPR*, were present in a founder. But in all extant insects and crustaceans that we examined, a Pol III-regulated *RPR* gene was not detected (12); instead, it seems that the new allele was fixed despite the added complexity of biogenesis from an intron. Although retention of the Pol II-regulated version and loss of the Pol III-regulated version might reflect an evolutionary event that happened by chance, it is also possible that the intronic RPR provided an advantage over the ancestral Pol III-regulated RPR gene. The ability to regulate RPR biogenesis through a multi-tiered integration of transcription, post-transcriptional processing, and RNP assembly may have favored this mode despite the added complexity.

## Supplementary Material

gkz572_Supplemental_File
