# Biogenesis of RNase P RNA from an intron requires co-assembly with cognate protein subunits

**DOI:** 10.1093/nar/gkz983

**Published:** 2019-10-18

**Authors:** Geeta Palsule, Venkat Gopalan, Amanda Simcox

**Affiliations:** 1 Department of Molecular Genetics, The Ohio State University, Columbus, OH 43210, USA; 2 Center for RNA Biology, The Ohio State University, Columbus, OH 43210, USA; 3 Department of Chemistry and Biochemistry, The Ohio State University, Columbus, OH 43210, USA

*Nucleic Acids Research*,2019, 47(16): 8746–8754, https://doi.org/10.1093/nar/gkz572

Some details regarding the construction of a plasmid in the supplementary methods, construction of plasmids and reporter genes, page iv, section #6, are incorrect. The vector, ‘*pU6-BbsI-chiRNA vector (Addgene # 45946)*’, should be ‘**pBlueScript (KS-)**’ and the restriction enzymes, ‘*EcoR*V and *Xba*I’, should be ‘***EcoR*I and *BamH*I**’.

CURRENT: 6. pMRP promoter-Dv RPR 5T4T (Supplementary Figure S5D): A DNA fragment corresponding to the D. virilis RPR (with the 5T4T mutations) under the control of the D. melanogaster MRP promoter was generated by GENEWIZ (see Supplementary Table S4 for sequence details). This fragment was cloned using recombination (InFusion cloning kit) into the linearized **pU6-BbsI-chiRNA** vector **(Addgene # 45946)** that had been digested with ***EcoR*V and *Xba*I**.

CORRECTION: 6. pMRP promoter-Dv RPR 5T4T (Supplementary Figure S5D): A DNA fragment corresponding to the D. virilis RPR (with the 5T4T mutations) under the control of the D. melanogaster MRP promoter was generated by GENEWIZ (see Supplementary Table S4 for sequence details). This fragment was cloned using recombination (InFusion cloning kit) into the linearized **pBlueScript (KS-)** vector that had been digested with ***EcoR*I and *BamH*I**.

The supplementary file has been updated.

