# Supplementary material for: Biogenesis of RNase P RNA from an intron requires co-assembly with cognate protein subunits
Source: Nucleic Acids Res. 2019 Jul 9;47(16):8746–54. doi: 10.1093/nar/gkz572 (PMC6797745; doi:10.1093/nar/gkz572)
Supplement: gkz572_Supplemental_File [file gkz572_supplemental_file.pdf]

SUPPLEMENTARY MATERIAL

Biogenesis of RNase P RNA from an intron requires co-assembly with cognate protein subunits

Geeta Palsule[1,3], Venkat Gopalan[2,3] and Amanda Simcox[1,3,*]

[1]Department of Molecular Genetics,

[2]Department of Chemistry and Biochemistry,

[3]Center for RNA Biology, The Ohio State University, Columbus, OH 43210

[*]Corresponding author: Amanda Simcox

(Correspondence: simcox.1@osu.edu)

SUPPLEMENTARY METHODS:

Construction of plasmids and reporter genes

Generation of the split-RFP reporter with native splice sites (Figure 1A) and the split-RFP reporter with non-functional splice-sites (Figure 2B) (1). All RFP-reporter genes generated here are modifications of split-RFP reporter in the pPacPL vector (Addgene) (1).

1. Split-RFP reporter with 'ideal' splice sites (Figure 1E): A DNA fragment containing RFP exons split by a *D. virilis* intron-containing RPR with the 'ideal', splice sites (5′ splice-site: 5′ GTTCGT 3′ changed to 5′ GTAAGT 3′; 3′ splice-site: 5′ ACAACCAATACAACAG 3′ mutated to 5′ TCTTTTTTTTCTTCAG 3′; (see Supplementary Table S4 for the whole sequence) was synthesized by GENEWIZ. This fragment was cloned using recombination (InFusion cloning kit) into pPacPL that had been digested with *Eco*RV and *Xba*I (NEB) to excise the RFP exons and wild type *D. virilis* intron.
2. Split-RFP reporter with 5′-intron deletion (Δ5′ Int) (Figure 2E): A DNA fragment containing RFP exons split by a *D. virilis* intron-containing RPR, with a 72-bp deletion in the 5′ intron sequence that leaves an intact splice site, was synthesized by GENEWIZ (see Supplementary Table S4 for the whole sequence). This fragment was cloned into the linearized split-RFP reporter using recombination (InFusion cloning kit), after the RFP exons and Dv intron were excised by digestion with *Eco*RV and *Xba*I.
3. U6-Dv RPR (Supplementary Figure S5A-2): This construct was generated by PCR amplification of the *D. virilis RPR,* using the '*Bbs*I *D. virilis* RPR forward primer' and

‘*Bam*HI *D. virilis* reverse primer’ (see Supplementary Table S5). The forward and reverse primers added *Bbs*I and *Bam*HI restriction sites, respectively, at the 5′- and 3′-termini of the Dv RPR amplicon. The PCR product was digested with *Bbs*I and *Bam*HI and cloned into the pU6-BbsI-chiRNA linearized vector (*Bbs*I- and *Bam*HI-digested) (Addgene # 45946) using ligation-based cloning.

4. pU6 Dv RPR with polyT mutations (5T4T) (Supplementary Figure S5A-3): *D. virilis* RPR has two polyT stretches (5T and 4T), which were mutated to match the corresponding sequence in *D. melanogaster* RPR that lacks the polyT stretches. Overlap PCR was used to introduce a mutation in the 4T stretch (T157C) in pU6-DV RPR using overlapping primers: ‘Dv RPR 4T-mutation Forward primer’ and ‘Dv RPR 4T-mutation Reverse primer’ (see Supplementary Table S5). The PCR reaction was treated with *Dpn*I (NEB) to digest parental plasmid DNA prior to transformation of chemically-competent *E. coli* DH5α. The resulting plasmid, with mutations in one stretch of polyT (4T), was used in a second round of overlap PCR to introduce two mutations in the 5T stretch (T53C and T55A) using primer pair ‘Dv RPR 5T-mutations Forward primer’ and ‘Dv RPR 5T-mutations Reverse primer’ (see Supplementary Table S5). The PCR reaction was treated with *Dpn*I (NEB) to digest parental plasmid DNA prior to transformation of chemically-competent *E. coli* DH5α.
5. The split-RFP reporter with polyT mutations (5T4T) (Figure 3C): The polyT stretches in the Dv RPR from the split-RFP reporter were mutated to match those in the Dv RPR 5T4T construct. PolyT mutations were engineered using “splicing by overlap extension (SOE)” PCR that spliced together three DNA fragments,

containing the RFP exons, split by *D. virilis* intron-containing RPR. The primer sequences to generate the three fragments are as follows (see Supplementary Table S5 for sequence details):

Fragment 1: '*Eco*RV forward primer' and Dv RPR 5T-mutations reverse primer; Fragment 2: 'Dv RPR 5T-mutations forward primer' and 'Dv RPR 4T-mutation reverse primer'; Fragment 3: 'Dv RPR 4T-mutation forward primer' and '*Xba*I reverse primer'. A single fragment (1.5 Kb) containing the RFP exons, split by the *D. virilis* intron-RPR containing polyT mutations, was generated using SOE-PCR that spliced together three DNA fragments using the *Eco*RV forward primer and the *Xba*I reverse primer (forward primer used for 'Fragment 1' and reverse primer used for 'Fragment 3', respectively (see Supplementary Table S5)). This fragment was cloned using recombination (InFusion cloning kit) into pPacPL that had been digested with *Eco*RV and *Xba*I to excise the RFP exons and wild type *D. virilis* intron.

6. pMRP promoter-Dv RPR 5T4T (Supplementary Figure S5D): A DNA fragment corresponding to the *D. virilis* RPR (with the 5T4T mutations) under the control of the *D. melanogaster* MRP promoter was generated by GENEWIZ (see Supplementary Table S4 for sequence details). This fragment was cloned using recombination (InFusion cloning kit) into the linearized pBlueScript (KS-) vector that had been digested with EcoRI and BamHI.

Legends for supplementary figures

Figure S1. Splicing and RPR maturation

(A) Depletion of Ldbr led to decrease in *D. melanogaster* mature RPR levels (quantitation of the northern blot in Figure 1B). (n=3)

(B, top) Split-RFP reporter. Act5C promoter (Pol II), grey; *RFP* exons, red; *Drosophila virilis* intron-encoding *RPR*; pink, RPR and intron-specific antisense probes indicated (See also Figure 1A; Supplementary Table S1). The size of the RPR, 3'- and 5'- introns is indicated. Intron, 701 nt; RPR, 353 nt. *In vitro*-transcribed RNAs corresponding to 701 nt (Intron + RPR), and 353 nt (RPR) were used as size markers.

(B, bottom) Northern blot of RNA extracted from S2 cells that were treated first with dsRNA to knockdown either lariat debranching enzyme, *Ldbr* (Ldbr KD) or GFP (control, GFP KD) and subsequently transfected with the split-RFP reporter (S1B, top). Hybridization with each of the indicated probes (1, 2, 3, 4, 5, 6 or 7) confirmed the presence of intron sequence in the intermediate. The intermediate was not detected with probe 3.

(C) RT-PCR to detect *Ldbr* knockdown (KD) efficiency using as input RNA extracted from S2 cells that were first treated with dsRNA to knockdown Ldbr or GFP and subsequently transfected with the split-RFP reporter. GAPDH was used as the loading control for RT-PCR (Related to Figure 1A and 1B).

(D) Histogram and accompanying table indicate raw splice-site scores for the 5′ donor and 3′ acceptor splice-sites (ss) for the first and second (*RPR*-containing) intron of the *ATPSynC* gene in 12 annotated *Drosophila* species. Zero indicates that a splice-site score was not predicted for that exon-intron junction (Related to Figure 1D).

(See Supplementary Table S1 for sequences of probes used, and Supplementary Tables S2 and S3 for sequences of primers used.)

Figure S2. The effect of nucleases on RPR processing

(A, top) Split-RFP reporter. Act5C-Pol II promoter, grey; *RFP* exons, red; *Drosophila virilis* intron (702 nt)-encoding *RPR* (353 nt), pink; RPR and intron specific antisense probes indicated (See also Figure 1A).

(A, bottom) Northern blot of RNA extracted from S2 cells that were first treated with dsRNA to knockdown either *Rat1/Xrn2* (Rat1 KD) or GFP (control, GFP KD) and subsequently transfected with the split-RFP reporter (S2A, top). The intermediate(s) in the Rat1/Xrn2-depleted sample were detected with the indicated probe (1, 2, 4, 5, or 6). (See also Figure 2C, which shows the intermediate also hybridized with probes 7 and 3).

(B) RT-PCR to determine the expression level of *Rat1, Dis3,* or *Rrp6* for RNA extracted from S2 cells that were treated with dsRNA to either the specified nuclease or GFP and subsequently transfected with the split-RFP reporter. PCR products were quantitated by ImageJ and normalized to GAPDH (data related to northern blots in Figures 2A and 2G).

(C) RT-PCR to determine the expression level of *Rat1* for RNA extracted from S2 cells treated with dsRNA to knockdown either Rat1 or GFP and subsequently transfected with the split-RFP reporter containing non-functional splice-sites. PCR products were quantitated by ImageJ and normalized to GAPDH. These RT-PCR data relate to the knockdown experiment depicted in Figure 2B.

(D-F, top) Northern blot of RNA extracted from S2 cells that were first treated with dsRNA to knockdown the indicated nuclease (*Rex2, IntS11* or *Rexo5*) or GFP and subsequently transfected with the split-RFP reporter (Supplementary Figure S2A). Hybridization with an antisense-RPR probe (Probe 1) showed that the RPR levels were unaffected. RT-PCR indicating knockdown of *Rai1*; these data relate to the knockdown experiment depicted in Figure 2A. (See Supplementary Table S1 for sequence of probe1 used for northern blot detection)

(D-F, bottom) Efficiency of knockdown for a given nuclease as determined by RT-PCR and compared to GFP. GAPDH was used as the loading control.

(G) Efficiency of knockdown for *Ldbr*, *Rat1* or *GFP* as determined by RT-qPCR. GAPDH was used as the normalization control. These data relate to the knockdown experiment in Figure 2F.

(H) RFP and phase-contrast images of S2 cells from which RNA was extracted for northern blot shown in Figure 2F. RFP expression was used to assess similar transfection efficiencies and splicing of RFP mRNA expressed from the split-RFP reporter with the 5' deletion (Figure 2E).

(See Supplementary Tables S2 and S3 for sequences of primers used for RT-qPCR and RT-PCR.)

Figure S3. Depletion of Rat1 or exosome nucleases led to decreased RPR levels

(A and B) Quantitation of the northern blot in Figure 2A shows that both *D. virilis* RPR (A) and *D. melanogaster* RPR (B) levels are decreased following depletion of Rat1 when compared to the GFP control. (n=3)

(C and D) Quantitation of the northern blot in Figure 2G shows that both *D. virilis* RPR (C) and *D. melanogaster* RPR (D) levels are decreased following depletion of Dis3 or Rrp6 when compared to the GFP control. (n=3)

Figure S4. Quantitation of Rpp knockdowns and RPR expression

(A-D) RT-qPCR quantitation to estimate the efficiency of knockdown of seven Rpps (*Pop1, Pop4, Rpp25, Rpp20, Pop5, Rpp14* and *Rpp30*) compared with GFP (control): RT-qPCR data in panels A, B, C and D relate to the experiments depicted in Figures 3A, 3B, 3C and 3F, respectively. (For Rpp21, see Supplementary Figure S4E).

(E) Gel electrophoresis of RT-qPCR products to estimate *Rpp21* knockdown in the indicated experiments. Rpp KD was efficient as confirmed by loss or decrease in level of the expected product (Rpp21). The additional product (indicated by ‘<’) might correspond to an amplicon produced from the pre-mRNA that contains the intron. Data for Rpp21 KD corresponding to Figures 3 A-C and F.

(F) Quantitation of *D. melanogaster* U6 snRNA-Pol III-transcribed *D. virilis* (Dv) RPR levels upon individual depletion of all eight Rpps: Pop1, Pop4, Rpp25, Rpp20, Pop5, Rpp14, Rpp30 or Rpp21. RPR levels were normalized to *D. melanogaster* U6 snRNA for loading. These data relate to the RPP knockdown experiment in Figure 3B. (n=2).

Figure S5. RPR expression from Pol II and Pol III promoters

(A) Reporter constructs encoding the *D. virilis RPR* expressed under regulation of the Act5C-Pol II promoter (1) or the U6 snRNA-Pol III promoter (2 and 3). The red asterisks in construct 3 indicate three point mutations (T53C, T55A, and T157C) that disrupt the two polythymidine (polyT) stretches within the *RPR* that could potentially act as Pol III

transcription termination signals. (See Supplementary Table S4 for details of sequences for promoters and mutated RPR gene)

(B) Northern blot of RNA extracted from S2 cells that were transfected with a given RPR reporter gene (1, 2 or 3, shown in Supplementary Figure S4A) or a 'no transfection' control. When expressed from a U6 snRNA-Pol III promoter, RPR was transcribed when the internal polyT stretches were mutated (construct 3, asterisk). IVT, *in vitro* transcription of the *D. virilis* intron-containing RPR (701 nt) and the *D. virilis* RPR (353 nt).

(C) RT-qPCR quantitation to estimate the efficiency of knockdown of *Rat1/Xrn2* and *Pop1* either singly or in combination; these data pertain to the northern blot shown in Figure 3E.

(D) Northern analysis of RNA extracted from S2 cells, treated with dsRNA to knockdown (KD) Pop1, Pop4 or GFP (control) and transfected with indicated constructs. *D. virilis* RPR transcribed by either the U6 snRNA- or MRP-Pol III promoters has mature termini and was unaffected when either Pop1 or Pop4 were depleted. This result indicates that the lack of dependence on Rpps during RPR biogenesis is not specific to a single Pol III promoter. The endogenous *D. melanogaster* RPR (Dm RPR) level is decreased upon depletion of Pop1 or Pop4, corroborating the data in Figures 3A-F. *D. melanogaster* U6 snRNA was used as the loading control.

Figure S6. Schematic indicating the locations of Pop1, Pop4, Rpp25 and Rpp20 in the high-resolution structure of human RNase P ((2); EMD-9626). This image was rendered using PyMol.

Figure S1

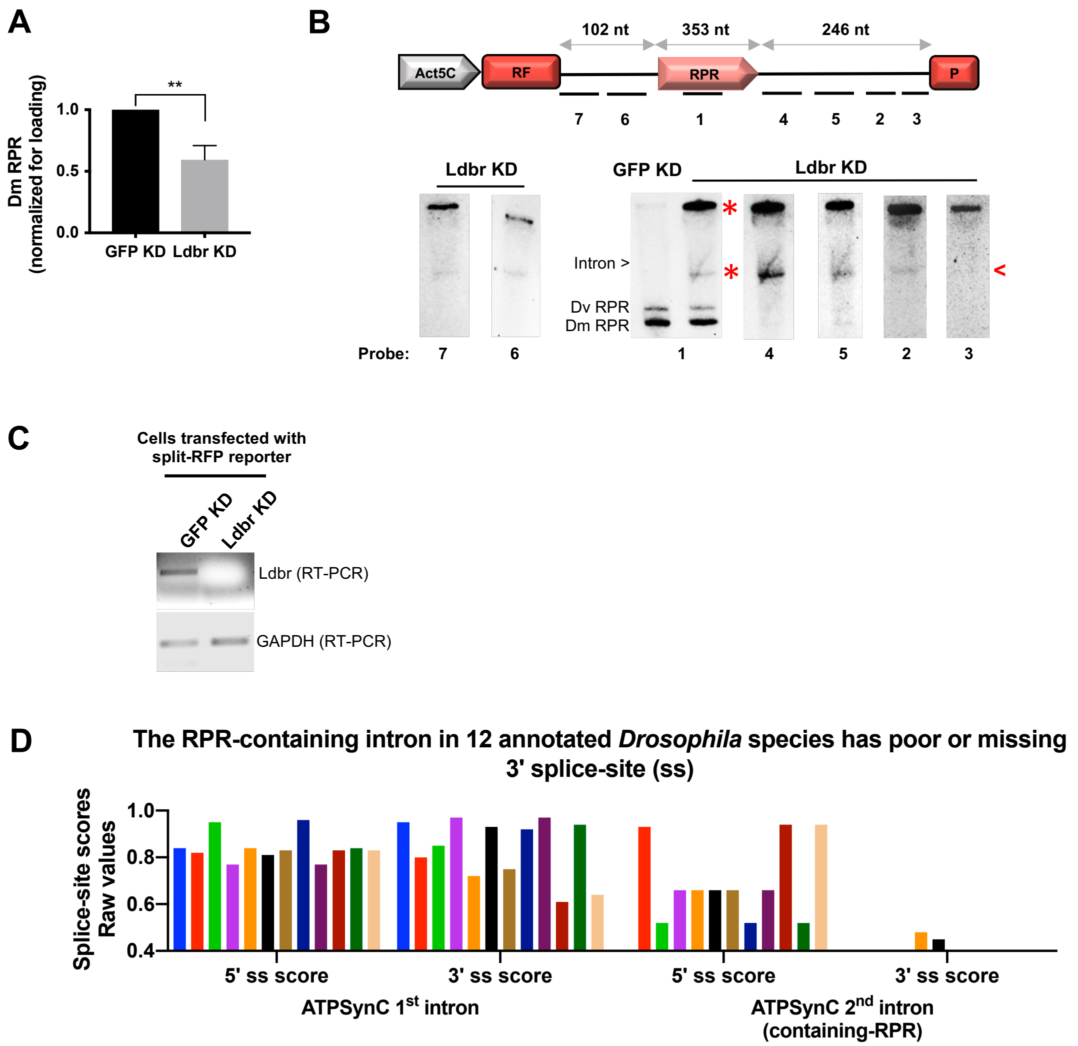

	ATPSynC 1st intron 5' ss score	ATPSynC 1st intron 3' ss score	ATPSynC 2nd intron 5' ss score	ATPSynC 2nd intron 3' ss score
D. virilis	0.84	0.95	0	0
D. melanogaster	0.82	0.8	0.93	0
D. grimshawi	0.95	0.85	0.52	0
D. pseudoobscura	0.77	0.97	0.66	0
D. yakuba	0.84	0.72	0.66	0
D. ananassae	0.81	0.93	0.66	0
D. erecta	0.83	0.75	0.66	0.48
D. mojavensis	0.96	0.92	0.52	0.45
D. persimilis	0.77	0.97	0.66	0
D. sechellia	0.83	0.61	0.94	0
D. willistoni	0.84	0.94	0.52	0
D. simulans	0.83	0.64	0.94	0

Figure S2

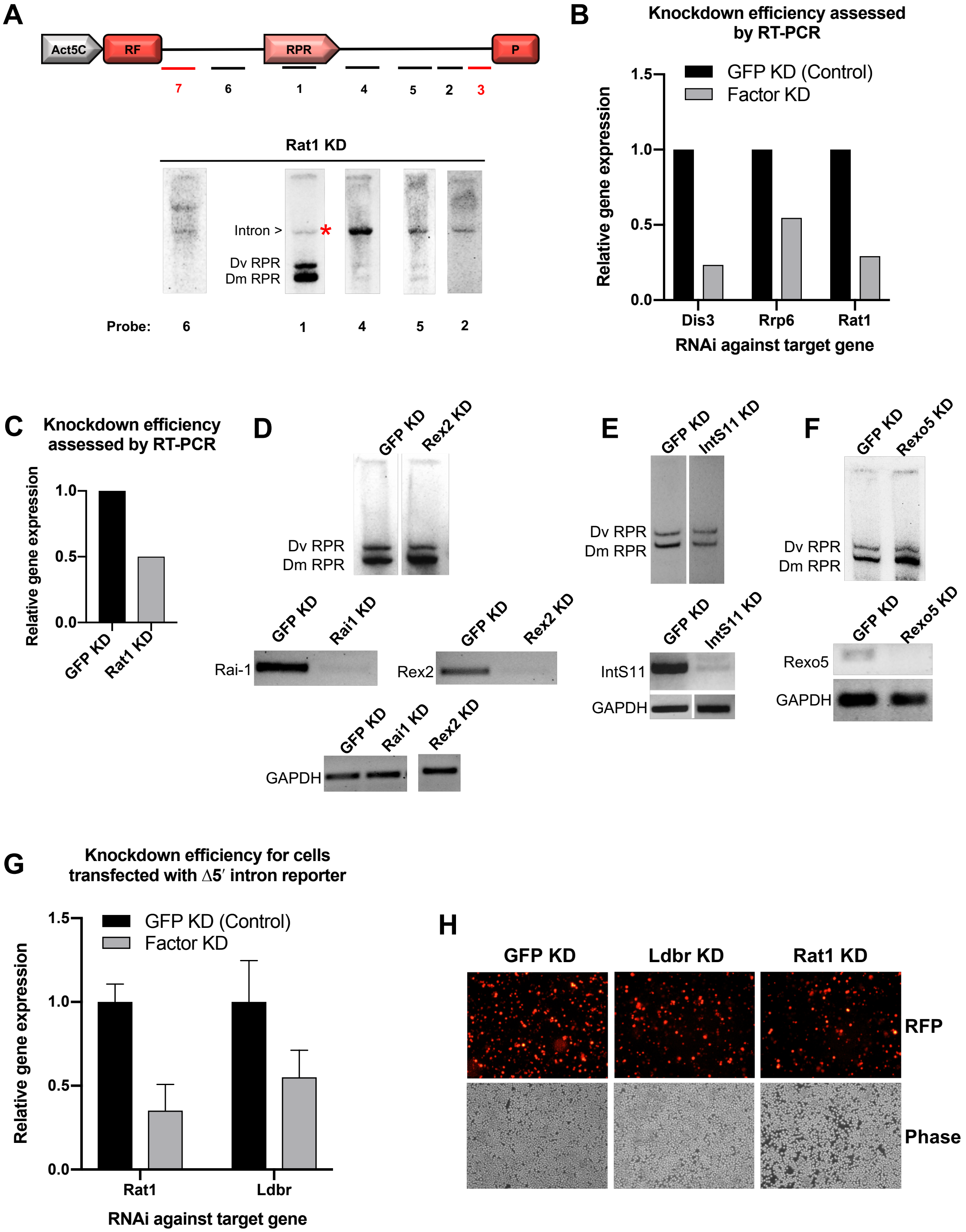
A
Act5C
RF
RPR
P
7
6
1
4
5
2
3
Rat1 KD
Intron >
*
Dv RPR
Dm RPR
Probe:
6
1
4
5
2
B
Knockdown efficiency assessed by RT-PCR
GFP KD (Control)
Factor KD
Relative gene expression
1.5
1.0
0.5
0.0
Dis3
Rrp6
Rat1
RNAi against target gene
C
Knockdown efficiency assessed by RT-PCR
Relative gene expression
1.0
0.5
0.0
GFP KD
Rat1 KD
D
GFP KD
Rex2 KD
Dv RPR
Dm RPR
GFP KD
Rai1 KD
Rai-1
GFP KD
Rex2 KD
Rex2
GFP KD
Rai1 KD
Rex2 KD
GAPDH
E
GFP KD
IntS11 KD
Dv RPR
Dm RPR
GFP KD
IntS11 KD
IntS11
GAPDH
F
GFP KD
Rexo5 KD
Dv RPR
Dm RPR
GFP KD
Rexo5 KD
Rexo5
GAPDH
G
Knockdown efficiency for cells transfected with Δ5′ intron reporter
GFP KD (Control)
Factor KD
Relative gene expression
1.5
1.0
0.5
0.0
Rat1
Ldbr
RNAi against target gene
H
GFP KD
Ldbr KD
Rat1 KD
RFP
Phase

Figure S3

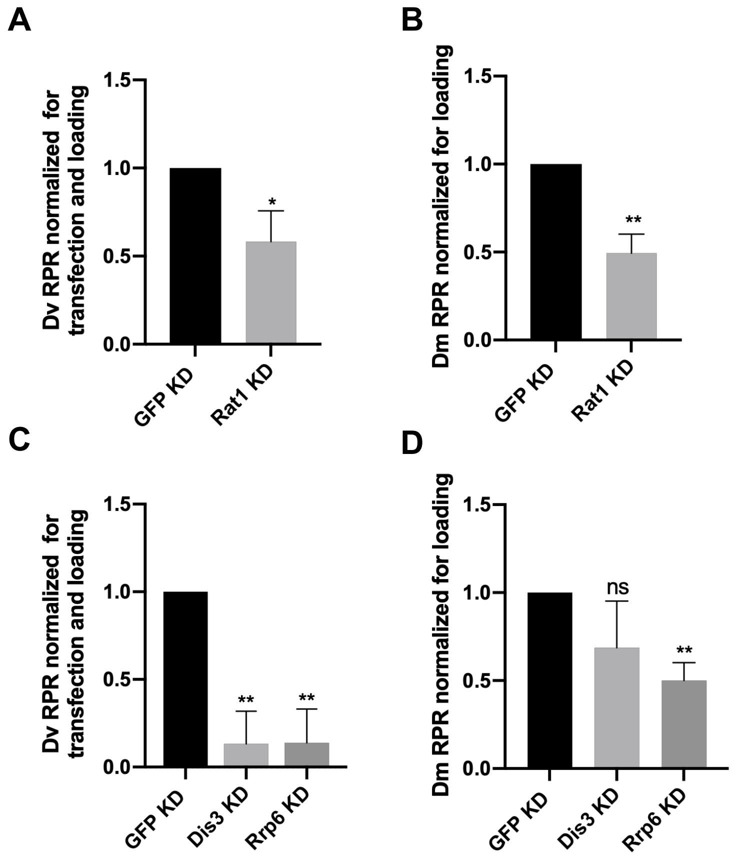
A
Dv RPR normalized for transfection and loading
1.5
1.0
0.5
0.0
*
GFP KD
Rat1 KD
B
Dm RPR normalized for loading
1.5
1.0
0.5
0.0
**
GFP KD
Rat1 KD
C
Dv RPR normalized for transfection and loading
1.5
1.0
0.5
0.0
**
**
GFP KD
Dis3 KD
Rrp6 KD
D
Dm RPR normalized for loading
1.5
1.0
0.5
0.0
ns
**
GFP KD
Dis3 KD
Rrp6 KD

Figure S4

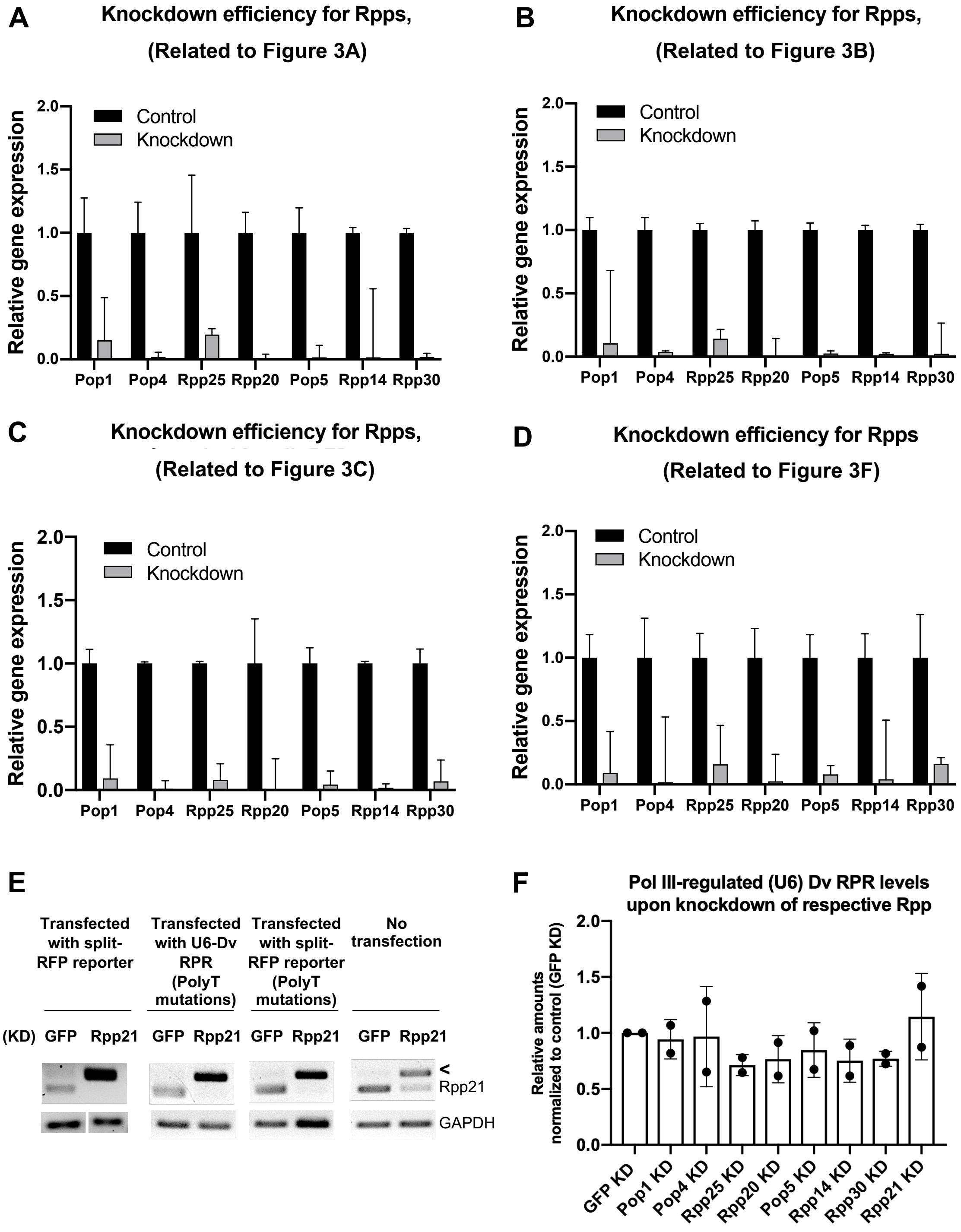
A
Knockdown efficiency for Rpps,
(Related to Figure 3A)
B
Knockdown efficiency for Rpps,
(Related to Figure 3B)
C
Knockdown efficiency for Rpps,
(Related to Figure 3C)
D
Knockdown efficiency for Rpps
(Related to Figure 3F)
Relative gene expression
Control
Knockdown
2.0
1.5
1.0
0.5
0.0
Pop1
Pop4
Rpp25
Rpp20
Pop5
Rpp14
Rpp30
E
Transfected with split-RFP reporter
Transfected with U6-Dv RPR (PolyT mutations)
Transfected with split-RFP reporter (PolyT mutations)
No transfection
(KD)
GFP
Rpp21
<
Rpp21
GAPDH
F
Pol III-regulated (U6) Dv RPR levels upon knockdown of respective Rpp
Relative amounts normalized to control (GFP KD)
GFP KD
Pop1 KD
Pop4 KD
Rpp25 KD
Rpp20 KD
Pop5 KD
Rpp14 KD
Rpp30 KD
Rpp21 KD

Figure S5

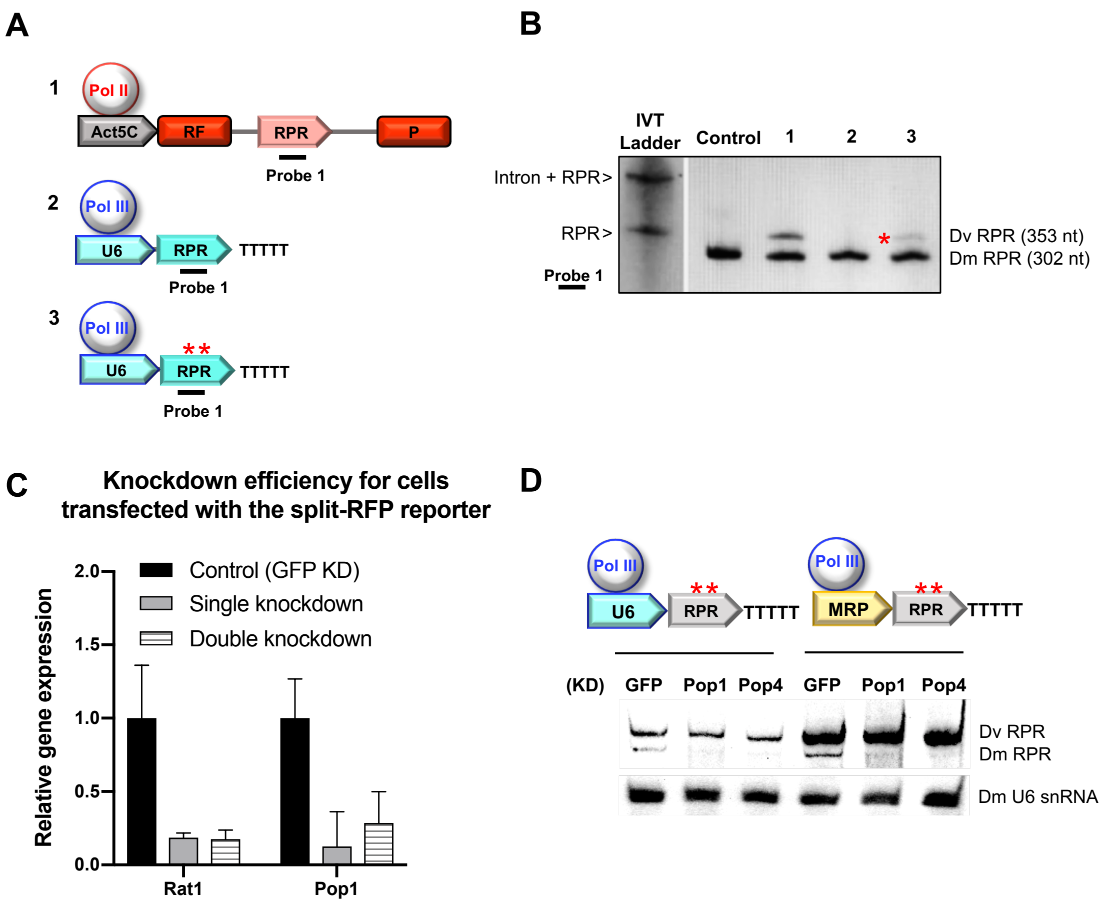
A
1
Pol II
Act5C
RF
RPR
P
Probe 1
2
Pol III
U6
RPR
TTTTT
Probe 1
3
Pol III
U6
RPR
TTTTT
Probe 1
B
IVT Ladder
Control
1
2
3
Intron + RPR>
RPR>
Probe 1
Dv RPR (353 nt)
Dm RPR (302 nt)
C
Knockdown efficiency for cells transfected with the split-RFP reporter
Relative gene expression
2.0
1.5
1.0
0.5
0.0
Control (GFP KD)
Single knockdown
Double knockdown
Rat1
Pop1
D
Pol III
U6
RPR
TTTTT
Pol III
MRP
RPR
TTTTT
(KD)
GFP
Pop1
Pop4
GFP
Pop1
Pop4
Dv RPR
Dm RPR
Dm U6 snRNA

Figure S6

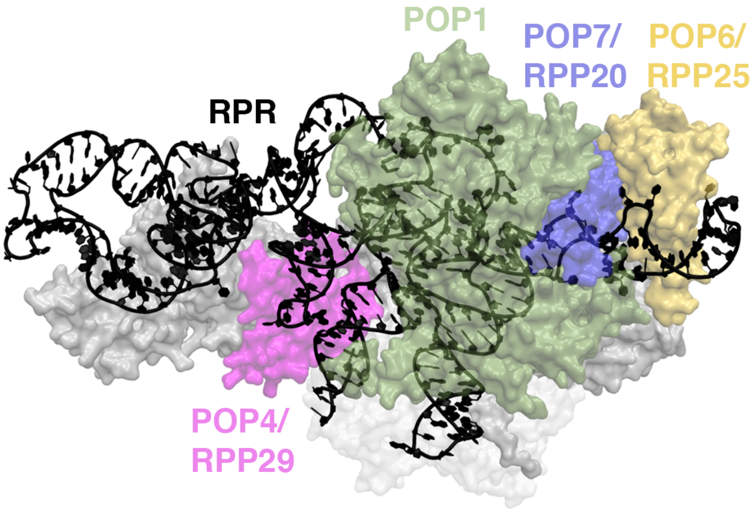

Legends for supplementary tables

Supplementary Table S1.

DNA probes used for northern hybridization. The probes are shown graphically in Figure 2A (probes 1-3) and Supplementary Figure S2A (probes 1-7).

Supplementary Table S2.

Primers used to generate gene-specific PCR products with T7 promoters used as templates for *in vitro* transcription (IVT) of dsRNAs for RNAi experiments.
PCR products for Rai1 and Rex2 were obtained directly from the DRSC (*Drosophila* RNAi Screening Center. There are two Rpp14 genes in *Drosophila, CG34317* (*) and *CG15526*. A dsDNA template was generated to target *CG34317*, which is expressed in S2 cells.

Supplementary Table S3.

Primer sequences used for PCR and qPCR to detect knockdown efficiency for the indicated gene.

Supplementary Table S4.

DNA sequence for the *RFP* exons, split by *D. virilis* intron-*RPR* and DNA fragments synthesized by Genewiz.

Supplementary Table S5.

Oligonucleotides used for construction of plasmids and reporter genes used in this study.

Table S1. DNA probes used for northern analysis (Figures 1-3, S1-S5)

DNA probes used in Northern analysis	Sequence (5'- 3')
D. virilis RPR (Probe 1)	CATACTGCAGCATTTCTGGTACTTTCGCCTTGTGATTACACAAGTTTCCGGCTAGAAAATGAAGCTCCGCGACACA CAATCACCTCTCGGCTTTTGTATGTTGTTACAGC
D. virilis RPR specific (Probe 1, Dv RPR specific)	CCGCGACACACAATCACCTCTCGGCTTTTGTATGTTGTTACAGCAACAAGGGAGCTTTGA
D. melanogaster RPR	AGGCGGCGGTCACCGGGGGCAACGGCAACACCTGGTAGCAGGATTTCTGGTACTTTCGCCCTGCAAT CACACAGGTTTCCTGCTGGGAAATGAAGCCGCAACAAACAGCT
D. melanogaster U6 snRNA	ACGCTTCACGATTTTGCGTGTCATCCTTGCGCAGGGGCCATGCTAATCTTCTCTGTATCGTTCCAATTTTAGTATAT GTTCTGCCGAAGC
D. virilis -3' Proximal intron (Probe 4)	TCCAACTCTGCCTGTTGGCATTTCTCATCTCTTTTATTGG
D. virilis -3' Distal intron (Probe 5)	GGCATATAGCTCTATATAGAGTTATATGCACAAAGCGATTTGG
D. virilis -3' Distal 1 intron (Probe 2)	ATTCGTACAGCAAATTGATATATATTGAAGTCGCATCGGC
D. virilis -3' Distal 2 intron (Probe 3)	GGTTGTCATTGTCATAGTAGCAAGCGCACATATATATACG
D. virilis -5' Proximal intron (Probe 6)	AGCGATGAATATAGCCGCTGGGCAGGATTGGACAAATTGA
D. virilis -5' Distal intron (Probe 7)	CATGGTAAGATGCTAATGTATCAGACAAGACGCTTAACAATTGG
$tRNA^{His}$ 5' leader probe	ACGGCTACATCGGGTGAT
$tRNA^{His}$ internal probe	GAACCTGGGTTACCACGGCCA

Table S2. Primers to create T7-dsDNA template for generation of dsRNA used for RNAi mediated knockdowns, related to main Figures 1-3, S1-S4

Gene Name (dsDNA length in bp)	Function or Flybase ID #	T7-Forward (5'-3')	T7-Reverse (5'-3')
GFP (609)	N/A	ATGCAGATATCTAATACGACTCACTATAGGG GACGTAAACGGCCACA	ATGCAGGATCCTAATACGACTCACTATAGGGGAACT CCAGCAGGAC
Ldbr (503)	Endonuclea se	TAATACGACTCACTATAGGGGAAACAAGGC GCA	TAATACGACTCACTATAGGGGTTTGATCGCTCTGTA
Rat1/Xrn2 (506)	5' to 3' exonuclease	ATGCAGATATCTAATACGACTCACTATAGGG TTATACAAGAAGTG	ATGCAGGATCCTAATACGACTCACTATAGGGCAGCC CTGATAGTAG
Dis3 (517)	3' to 5 exonuclease	ATGCAGATATCTAATACGACTCACTATAGGG ATCATCGTAACGATTG	ATGCAGGATCCTAATACGACTCACTATAGGGCTTCA TTGTCCACTTC
Rrp6 (508)	3' to 5' exonuclease	ATGCAGGATCCTAATACGACTCACTATAGGG TTATCGTTGTATATCG	ATGCAGATATCTAATACGACTCACTATAGGGGCATC TCCCTTGGAAG
Rai1 (492)	5' to 3' exonuclease	DRSC20157	N/A
IntS11 (490)	Endonuclea se	TAATACGACTCACTATAGGGGTGTCGCGAG AGG	TAATACGACTCACTATAGGGGACCACCTGGCG
Rexo5 (497)	3' to 5' exonuclease	TAA TAC GAC TCA CTA TAG GGG GGCACGCAGACTGATAGA	TAA TAC GAC TCA CTA TAG GGG ACACTTCCTTTTGCACAACAT
Rex2 (496)	3' to 5' exonuclease	DRSC14213	N/A
Pop1 (489)	CG32763	TAA TAC GAC TCA CTA TAG GGCAGCGGGAGTTTAATCGTT	TAA TAC GAC TCA CTA TAG GGGTAGCCCAATCTCTTGTATCT
Pop4/Rpp29 (516)	CG8038	TAA TAC GAC TCA CTA TAG GGGACCAGCGTTGCAATGTTAA	TAA TAC GAC TCA CTA TAG GGGCGGGTCGAATGTTGAGGT
Rpp25/Pop6 (202)	CG9422	TAA TAC GAC TCA CTA TAG GGGGCCAAATACGTCCACTGATT	TAA TAC GAC TCA CTA TAG GGGACTGGCGGGTAAATTCTCT
Rpp20/Pop7 (536)	CG33931	TAA TAC GAC TCA CTA TAG GGGCGCCCACGAGATCTTTCT	TAA TAC GAC TCA CTA TAG GGGGACGCAAAAGTACGCGTT
Pop5 (216)	CG14057	TAA TAC GAC TCA CTA TAG GGGTGCCCTACACGCCAACA	TAA TAC GAC TCA CTA TAG GGGAAGTAATCAATGGCAATACGC
Rpp14*/Pop8 (445)	CG34317	TAA TAC GAC TCA CTA TAG GGGGGCAAGGACAAGGTGATTTTT	TAA TAC GAC TCA CTA TAG GGGCCTTGGGCACCACTGAAC
Rpp30/Rpp1 (254)	CG11606	TAA TAC GAC TCA CTA TAG GGGATGGGGACTTGATAACTTTTGA	TAA TAC GAC TCA CTA TAG GGGATTAGCAACGTCGTAAGGTC
Rpp21/Rpr2 (539)	CG33082	TAA TAC GAC TCA CTA TAG GGGCATCTCCTCACGCATGAAC	TAA TAC GAC TCA CTA TAG GGGACCACTTGCACTATGGATTG

Table S3. Primers to detect knockdown efficiency with PCR or qPCR, related to main Figures 1-3

Gene name (amplicon size in bp)	Forward (5'-3')	Reverse (5'-3')
Ldbr (252)*	AGCTGTCGAAGGATGTGCACA	GCCTCATGGTTGCCGCC
Ldbr (86)	CGAGGAAAGGGAGAAGGTAAAG	TGTCGAGAGCCAGAAACTTG
Rat1/Xrn2 (250)*	CCGGATAGTGGTCACCGCATG	TGTGGCCATATTGCTGCTGCT
Rat1/Xrn2 (110)	TGCTGAACGCTGGCATAA	TCCTTGGTTTCCGCCTATTC
Dis3 (193)*	AGCTATTGGACCGAAAGTCAAATGAGGA	CCATACTTGGGGATTAGCACTTGCA
Rrp6 (250)*	GCACAGAACAACCGCAAGCAAAA	GCTTGTTGAACTGCTTGTTGTTCCGATTG
Rai1 (250)*	GCCGGTCAACGAGGCAG	GCCCACCAGGAATGACTGA
IntS11 (246)*	CCGTGGGCAACAAGATTCTCG	TGGCCGGCATGTAGGTTTCC
Rexo5 (146)*	AGCACGAAGCTGGAGATGATAC	TTGTTCAGCTCCACAGCCGA
Rex2/CG10214 (250)*	ATGCTTGCTCACCTTCGACG	CCTCCTGCGGATGGTTGATG
Pop1 (150)	GGATTGAGGGAACTGGATTCGG	GTAATTGGTACGCTTATTGGGCGG
Pop4/Rpp29 (150)	GAGGGAATTTATCTCAGATCTGGTGATTCC	CGGCGACTCAGCGTACTGG
Rpp25/Pop6 (107)	GTCACACGCCAAATACCCAC	CCATCCCAAAAATCAGTGGACGTA
Rpp20/Pop7 (146)	CCGCGTCCCGCTGTTA	CCGCGGGTTACGGAGAA
Pop5 (150)	GCAATCATACGCTGTCTCCATCG	TTGGTGCTTGACTATGAACTTGTTGC
Rpp14/Pop8 (138)	GAGTGGTTACCAATATCTGGACGTG	AGTATGGTCTGGCCACCTATTTCG
Rpp30/Rpp1 (150)	GGAGCAAACAAGGCCATTTTACG	TTGCCCGGATCCTTTTTGCTGT
Rpp21/Rpr2 (110)	CCGCCTATTATGGCAAACTCTGC	GGGAATCAGGGGAAGGAAACA
GAPDH (110)*	TGGCCGTCAACGATCCC	GAATCCGCCCTCAGCC
RFP (155)*	AGTGGGAGCGCGTGATGA	CCAGCCCATAGTCTTCTTCTGCAT
RFP (91)	AGGACGGCTGCTTCATCTA	TCCCAGCCCATAGTCTTCTT

* Indicates primers used for KD detection by PCR, rest used for qPCR mediated KD detection. GAPDH primers were used for both, PCR and qPCR

Table S4. Synthetic DNA fragments used for cloning (Figures 1-3 and S5, See also Method details) (Red, RFP exon; purple, Dv RPR; intron, lower case)

**DNA fragment name (*synthesized by GENEWIZ)**	**Sequence (5'-3')**
Split-RFP reporter containing *D. virilis* intron+ RPR with native splice-sites	ATGGCCTCCTCCGAGGACGTCATCAAGGAGTTCATGCGCTTCAAGGTGCGCATGGAGGGCTCCGTGAACG<br>GCCACGAGTTCGAGATCGAGGGCGAGGGCGAGGGCCGCCCCTACGAGGGCACCCAGACCGCCAAGCTGA<br>AGGTGACCAAGGGCGGCCCCCTGCCCTTCGCCTGGGACATCCTGTCCCCCCAGTTCCAGTACGGCTCCAA<br>GGTGTACGTGAAGCACCCCGCCGACATCCCCGACTACAAGAAGCTGTCCTTCCCCGAGGGCTTCAAGTGG<br>GAGCGCGTGATGAACTTCGAGGACGGCGGCGTGGTGACCGTGACCCAGGACTCCTCCCTGCAGGACGGC<br>TGCTTCATCTACAAGgttcgtattggttaccaattgttaagcgtcttgtctgatacattagcatcttaccatgattcaatttgtccaatcctgcccagcggcta<br>tattcatcgcttcAGTCAGTTACAATCTAGCATCTGGGGCACACACAACGAGTATCTGATTACTCTTTTTACAACGCC<br>CCGGGAAGGTCTGAGATATGGTCAAAGCTCCCTTGTTGCTGTAACAACATACAAAAGCCGAGAGGTGATTG<br>TGTGTCGCGGAGCTTCATTTTCTAGCCGGAAACTTGTGTAATCACAAGGCGAAAGTACCAGAAATGCTGCAG<br>TATGGCTGTTGCCGCACCGCTTGCCGTTTGCCGCCGGCCATGTTGCATTGAAAACTTTCGTGACCAGCAATT<br>TAAGTGCGATGTGCTTGCTGCAAACCTTGCCTGAGGTGCGCAGAACTCAATTCAGACTAATCTGTGACTGAC<br>Tggcccaataaaagagatgagaaatgccaacaggcagagttggattttgattcaaatccaaatcgctttgtgcatataactctatatagagctatatgcctatca<br>tccgtcggcaattgccgatgcgacttcaatatatatcaatttgctgtacgaattaattaatctattttgtctctttttctcaacatttttctcgtatatatatgtgcgcttgctac<br>tatgacaatgacaaccaatacaacagGTGAAGTTCATCGGCGTGAACTTCCCCTCCGACGGCCCCGTAATGCAGAAGA<br>AGACTATGGGCTGGGAGCCCTCCACCGAGCGCCTGTACCCCCGCGACGGCGTGCTGAAGGGCGAGATCC<br>ACAAGGCCCTGAAGCTGAAGGACGGCGGCCACTACCTGGTGGAGTTCAAGTCCATCTACATGGCCAAGAA<br>GCCCGTGCAGCTGCCCGGCTACTACTACGTGGACTCCAAGCTGGACATCACCTCCCACAACGAGGACTACA<br>CCATCGTGGAGCAGTACGAGCGCGCCGAGGGCCGCCACCACCTGTTCCTGTAG
Split-RFP reporter containing *D. virilis* intron+ RPR with 'ideal' splice-site mutations (underlined) (1414 bp)*	GACCCCGGATCCGATATCATGGCCTCCTCCGAGGACGTCATCAAGGAGTTCATGCGCTTCAAGGTGCGCAT<br>GGAGGGCTCCGTGAACGGCCACGAGTTCGAGATCGAGGGCGAGGGCGAGGGCCGCCCCTACGAGGGCA<br>CCCAGACCGCCAAGCTGAAGGTGACCAAGGGCGGCCCCCTGCCCTTCGCCTGGGACATCCTGTCCCCCCA<br>GTTCCAGTACGGCTCCAAGGTGTACGTGAAGCACCCCGCCGACATCCCCGACTACAAGAAGCTGTCCTTCC<br>CCGAGGGCTTCAAGTGGGAGCGCGTGATGAACTTCGAGGACGGCGGCGTGGTGACCGTGACCCAGGACT<br>CCTCCCTGCAGGACGGCTGCTTCATCTACAAGgtaagtattggttaccaattgttaagcgtcttgtctgatacattagcatcttaccatgattc<br>aatttgtccaatcctgcccagcggctatattcatcgcttCAGTCAGTTACAATCTAGCATCTGGGGCACACACAACGAGTATCTGA<br>TTACTCTTTTTACAACGCCCCGGGAAGGTCTGAGATATGGTCAAAGCTCCCTTGTTGCTGTAACAACATACAA<br>AAGCCGAGAGGTGATTGTGTGTCGCGGAGCTTCATTTTCTAGCCGGAAACTTGTGTAATCACAAGGCGAAA<br>GTACCAGAAATGCTGCAGTATGGCTGTTGCCGCACCGCTTGCCGTTTGCCGCCGGCCATGTTGCATTGAAA<br>ACTTTCGTGACCAGCAATTTAAGTGCGATGTGCTTGCTGCAAACCTTGCCTGAGGTGCGCAGAACTCAATTC<br>AGACTAATCTGTGACTGACTggcccaataaaagagatgagaaatgccaacaggcagagttggattttgattcaaatccaaatcgctttgtgcata<br>taactctatatagagctatatgcctatcatccgtcggcaattgccgatgcgacttcaatatatatcaatttgctgtacgaattaattaatctattttgtctctttttctcaac<br>atttttctcgtatatatatgtgcgcttgctactatgacaatgtctttttttcttcagGTGAAGTTCATCGGCGTGAACTTCCCCTCCGACGGCC<br>CCGTAATGCAGAAGAAGACTATGGGCTGGGAGCCCTCCACCGAGCGCCTGTACCCCCGCGACGGCGTGCT<br>GAAGGGCGAGATCCACAAGGCCCTGAAGCTGAAGGACGGCGGCCACTACCTGGTGGAGTTCAAGTCCATC<br>TACATGGCCAAGAAGCCCGTGCAGCTGCCCGGCTACTACTACGTGGACTCCAAGCTGGACATCACCTCCCA<br>CAACGAGGACTACACCATCGTGGAGCAGTACGAGCGCGCCGAGGGCCGCCACCACCTGTTCCTGTAGTCT<br>AGAGGTACCGAGCT
Split-RFP reporter containing *D. virilis* intron+ RPR with 5′ intron deletion (Δ5′ Intron) (1342 bp)*	GACCCCGGATCCGATATCATGGCCTCCTCCGAGGACGTCATCAAGGAGTTCATGCGCTTCAAGGTGCGCAT<br>GGAGGGCTCCGTGAACGGCCACGAGTTCGAGATCGAGGGCGAGGGCGAGGGCCGCCCCTACGAGGGCA<br>CCCAGACCGCCAAGCTGAAGGTGACCAAGGGCGGCCCCCTGCCCTTCGCCTGGGACATCCTGTCCCCCCA<br>GTTCCAGTACGGCTCCAAGGTGTACGTGAAGCACCCCGCCGACATCCCCGACTACAAGAAGCTGTCCTTCC<br>CCGAGGGCTTCAAGTGGGAGCGCGTGATGAACTTCGAGGACGGCGGCGTGGTGACCGTGACCCAGGACT<br>CCTCCCTGCAGGACGGCTGCTTCATCTACAAGgttcgtattggcggctatattcatcgcttcAGTCAGTTACAATCTAGCATC<br>TGGGGCACACACAACGAGTATCTGATTACTCTTTTTACAACGCCCCGGGAAGGTCTGAGATATGGTCAAAGC<br>TCCCTTGTTGCTGTAACAACATACAAAAGCCGAGAGGTGATTGTGTGTCGCGGAGCTTCATTTTCTAGCCGG<br>AAACTTGTGTAATCACAAGGCGAAAGTACCAGAAATGCTGCAGTATGGCTGTTGCCGCACCGCTTGCCGTTT<br>GCCGCCGGCCATGTTGCATTGAAAACTTTCGTGACCAGCAATTTAAGTGCGATGTGCTTGCTGCAAACCTTG<br>CCTGAGGTGCGCAGAACTCAATTCAGACTAATCTGTGACTGACTggcccaataaaagagatgagaaatgccaacaggcaga<br>gttggattttgattcaaatccaaatcgctttgtgcatataactctatatagagctatatgcctatcatccgtcggcaattgccgatgcgacttcaatatatatcaatttgc<br>tgtacgaattaattaatctattttgtctctttttctcaacatttttctcgtatatatatgtgcgcttgctactatgacaatgacaaccaatacaacagGTGAAGTTCA<br>TCGGCGTGAACTTCCCCTCCGACGGCCCCGTAATGCAGAAGAAGACTATGGGCTGGGAGCCCTCCACCGA<br>GCGCCTGTACCCCCGCGACGGCGTGCTGAAGGGCGAGATCCACAAGGCCCTGAAGCTGAAGGACGGCGG<br>CCACTACCTGGTGGAGTTCAAGTCCATCTACATGGCCAAGAAGCCCGTGCAGCTGCCCGGCTACTACTACG<br>TGGACTCCAAGCTGGACATCACCTCCCACAACGAGGACTACACCATCGTGGAGCAGTACGAGCGCGCCGA<br>GGGCCGCCACCACCTGTTCCTGTAGTCTAGAGGTACCGAGCT
D. melanogaster MRP promoter (bold)- *D. virilis* RPR with mutations (bold, underlined) in polyT stretches (685bp)*	GCTTGATATCGAATTC**CTGCAATCAAAAATGGGAAATGTATATTTAATAAAATACACAAAAACATGTGGTTC**<br>**ACATAGATCTAAAAATATTTAATGAGTTCGCCCCGCGCGGGTTTTGAACCCGTATCTTTCGGATTGGGGTG**<br>**TGCTATTCATATCGCGTTCGGCTGGTTATGATTCCCAACTCGTTTTTCCGCTGATGGCGCGTATAAATAGCG**<br>**CTGATAGCAGCAAATGTGATG**AGTCAGTTACAATCTAGCATCTGGGGCACACACAACGAGTATCTGATTACT<br>C**C**T**A**TTACAACGCCCCGGGAAGGTCTGAGATATGGTCAAAGCTCCCTTGTTGCTGTAACAACATACAAAAGC<br>CGAGAGGTGATTGTGTGTCGCGGAGCTTCATTT**C**CTAGCCGGAAACTTGTGTAATCACAAGGCGAAAGTAC<br>CAGAAATGCTGCAGTATGGCTGTTGCCGCACCGCTTGCCGTTTGCCGCCGGCCATGTTGCATTGAAAACTTT<br>CGTGACCAGCAATTTAAGTGCGATGTGCTTGCTGCAAACCTTGCCTGAGGTGCGCAGAACTCAATTCAGACT<br>AATCTGTGACTGACTTTTTTTATACAACGATATCATAACATATATATATATAACCCCCACCCCCCTTGGAATCA<br>TGATCTTTATACTTTACTCACGGATCCACTAGTTCTA

Table S5. Primers used for generation of plasmids and reporter constructs (underlined nucleotides represent mutations) (Figures 1-3)

Primer name	Sequence (5'-3')
*Bbs*I *D. virilis* RPR forward primer	TAACATGAAGACCACTTCGGAGTCAGTTACAATCTAG
*Bam*HI *D. virilis* reverse primer	ATGTTAGGATCCAAAAAAGTCAGTCACAGATTAGT
Dv RPR 4T-mutation forward primer	TGTGTGTCGCGGAGCTTCATTTCCTAGCCGGAAACTTGTGTA
Dv RPR 4T-mutation reverse primer	TACACAAGTTTCCGGCTAGGAAATGAAGCTCCGCGACACACA
Dv RPR 5T-mutations forward primer	CACAACGAGTATCTGATTACTCCTATTACAACGCCCCGGGAAGG
Dv RPR 5T-mutations reverse primer	CCTTCCCGGGGCGTTGTAATAGGAGTAATCAGATACTCG TTGTG
*Eco*RV forward primer	GACCCCGG ATCCGATATCATGGCCTCCTCCGAGGACG
*Xba*I reverse primer	GCTCGGTACCTCTAGACTACAGGAACAGGTGGTGGCG

References

1. Manivannan, S.N., Lai, L.B., Gopalan, V. and Simcox, A. (2015) Transcriptional control of an essential ribozyme in Drosophila reveals an ancient evolutionary divide in animals. *PLoS Genet*, **11**, e1004893.
2. Wu, J., Niu, S., Tan, M., Huang, C., Li, M., Song, Y., Wang, Q., Chen, J., Shi, S., Lan, P. *et al.* (2018) Cryo-EM structure of the human ribonuclease P holoenzyme. *Cell*, **175**, 1393-1404 e1311.
